# Antibacterial activity of green synthesized copper oxide nanoparticles against multidrug-resistant bacteria

**DOI:** 10.1038/s41598-024-75147-0

**Published:** 2024-10-23

**Authors:** Toka Khairy, Dina Hatem Amin, Hanaa Mohamed Salama, Iman Mohamed Amin Elkholy, Mostafa Elnakib, Hassan Mahmoud Gebreel, Hayam Abd Elnabi Sayed

**Affiliations:** 1 Department of Microbiology, Faculty of Science, Ain Shams University, El- Khalyfa El-Mamoun Street, Abbasya, Cairo Egypt; 2 Department of Chemistry, Faculty of Science, Port Said University, Port Said, 42521 Egypt; 3 Ain Shams Specialized Hospital, Ain Shams University, El-Khalyfa El-Mamoun Street, Abbasya, Cairo Egypt; 4 Medical Microbiology and Immunology, Military Medical Academy, Ehsan Abdelkodos Street, Manshyt Elbakry, Ciro, Egypt

**Keywords:** Copper oxide nanoparticle, Healthcare-associated infection, Plant extract, Antibacterial activity, Multidrug-resistant bacteria, Microbiology, Nanoscience and technology

## Abstract

Using plant extracts in the green synthesis of nanoparticles has become an environmentally acceptable approach. In our study, copper oxide nanoparticles (CuO NPs) were synthesized using ethanolic extracts of *Azadirachta indica* and *Simmondsia chinensis*. CuO NP formation was confirmed by the change in color and by UV‒visible spectroscopy (CuO NPs peaked at a wavelength of 344 nm). TEM images confirmed the semispherical shape of the CuO NPs, with particle sizes ranging from 30.9 to 10.7 nm. The antibacterial activity of these NPs was evaluated by using the agar diffusion method against clinical isolates, including methicillin-resistant *Staphylococcus aureus* (MRSA), *Escherichia coli*, *Pseudomonas aeruginosa*, *Acinetobacter* spp., *Klebsiella pneumoniae*, and *Stenotrophomonas maltophilia*. The minimum inhibitory concentration (MIC) of CuO NPs ranged from 62.5 to 125 µg/ml. In contrast, the antioxidant activity and antibiofilm activity of CuO NPs ranged from 31.1 to 92.2% at 125–500 µg/ml and 62.2–95%, respectively, at 125 –62.5 µg/ml. Our results confirmed that CuO NPs had IC50s of 383.41 ± 3.4 and 402.73 ± 1.86 at 250 µg/mL against the HBF4 cell line. Molecular docking studies with CuO NPs suggested that penicillin-binding protein 4 (PBP4) and beta-lactamase proteins (OXA-48) strongly bind to *S. aureus* and *K. pneumoniae*, respectively, with CuO NPs. Our study confirms the promising use of CuO NPs in treating pathogenic bacteria and that CuO NPs could be possible alternative antibiotics. This study supports the pharmaceutical and healthcare sectors in Egypt and worldwide.

**Supplementary Information:**

The online version contains supplementary material available at 10.1038/s41598-024-75147-0.

## Introduction

The effectiveness of standard antibiotics against common bacterial infections is being threatened by the increase in antibiotic resistance worldwide. The alarming rates of resistance among common bacterial infections are highlighted in the 2022 Global Antimicrobial Resistance and Use Surveillance System (GLASS) report^1^.

Multidrug-resistant (MDR) bacterial infections are primarily acquired in healthcare settings in high-income countries^2,3^. Limiting outbreaks requires identifying efficient infection management strategies to prevent MDR microbes from spreading from patients to the environment. According to recent publications, regulations about contact isolation and/or specific rooms for patients who are colonized or infected may not provide any significant benefits compared to routine infection control measures^4,5^. Many bacterial strains have become more resistant as a result of conventional medicine. In addition, patients who have taken these antibiotics at higher doses and for longer periods have experienced hepatic and renal toxicities in addition to persistent bacterial infections^6^. The creation of strong techniques with fewer harmful effects is necessary to overcome the negative effects of antibiotics and lower the incidence of bacterial infections, such as those caused by the biosynthesis of nanoparticles by using plants. Recently, nanotechnology has advanced tremendously in terms of scientific study and applications^7,8^. Recent advances in nanotechnology have led to the creation of nanoscale particles with potent antibacterial activity against multidrug resistant bacterial pathogens^9^. Metal NPs can be divided into numerous categories, such as copper and silver^10,11^. Nonetheless, it’s crucial to discuss how nanoparticles will affect the environment. Depending on several variables, including concentration, size, and duration of exposure, nanoparticles may be harmful to humans, animals, and plants, according to certain studies. It is easy for nanoparticles to be released into the environment through various factors, including consumer products, medical procedures, and industrial pollutants. Once released into the environment, nanoparticles can be difficult to control and monitor. Nanoparticles have the potential to persist for extended periods of time and can accumulate in the environment. This gives rise to worries over the possibility that nanoparticles may harm human health and the environment in the long run. However, controlling the synthesis process is one way to lessen the impact on the environment^12^.

Recently, green nanotechnologies have been found to be important in a range of biomedical fields. Natural reagents, plant extracts, reductants and capping agents can be used as effective substitutes for chemical and physical procedures in the production of nanoparticles^13^. Green synthesis has replaced conventional techniques, but one major obstacle is the lack of information regarding its effects on human health and the environment. By using natural extracts instead of chemical components, expecting to contribute with less environmental impacts^14^.

Compared with other analogous approaches, the green synthesis of nanoparticles employing live cells and biological pathways is more efficient and yields a larger mass. Many constituents and biochemical agents that can function as stabilizing and reducing agents to synthesize green nanoparticles can be found in plants. Compared with alternative biological, physical, and chemical approaches, green synthesis methods are more stable, nontoxic, economical, and environmentally benign^15^. Therefore, we are interested in studying the biogenic synthesis of copper nanoparticles from plant extracts. Plants have been extensively investigated because of their important nutritional value, beneficial phytochemicals, and low negative effects^16–18^. They contain a broad spectrum of bioactive substances with antibacterial, antifungal, anticancer, anti-inflammatory, and antioxidant properties^19–21^.

*Azadirachta indica*, commonly known as the Neem tree, is a plant that is native to India and has spread throughout most tropical and subtropical regions of the world. It is highly valued for its therapeutic properties^22^. It belongs to the family *Meliacea*. This plant has been shown to have antibacterial activity against both Gram-negative and Gram-positive bacteria^23^. It is also active against drug-resistant infections, which makes it a potential antibiotic^24^. Numerous physiologically active substances, such as alkaloids, flavonoids, terpenoids, phenolic compounds, carotenoids, steroids, and ketones, are present in the chemical elements of Neem^25^.

**Fig. 1:**
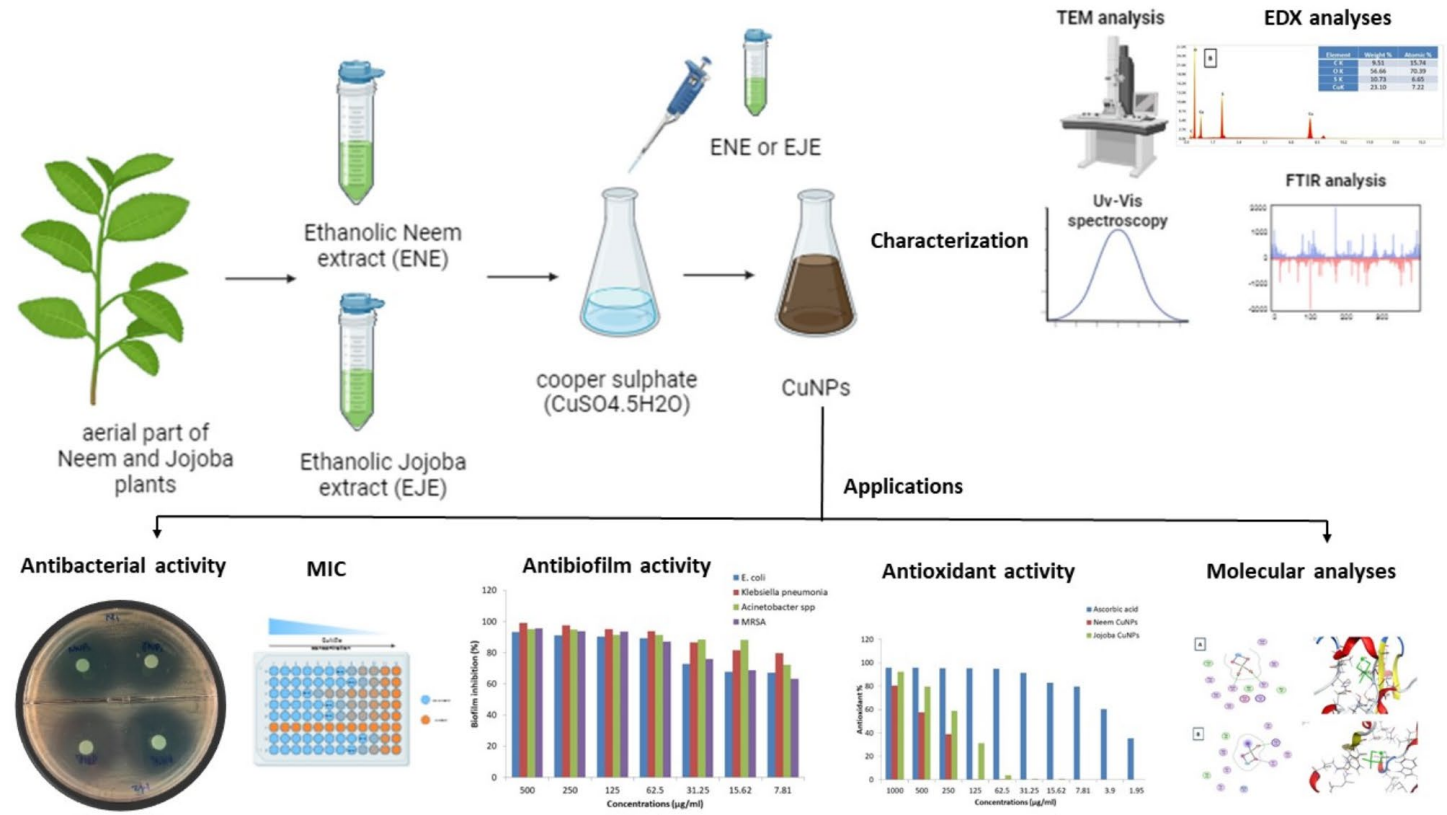
General outline of the work performed in this study.

*Simmondsia chinensis*, or Jojoba, is a member of the *Simmondsiaceae* family. The majority are woody perennial shrubs that are evergreen and yield tiny seeds that are filled with a waxy liquid^26^. The plant is native to southern Arizona, Sonora and Baja California. Recently, it has been cultivated around the world, including in Egypt, because of its considerable economic value^27^. Jojoba extracts showed sig nificant anti-bacterial properties; *Escherichia coli*,* Staphylococcus aureus*,* Bacillus cereus*,* Listeria monocytogenes and Salmonella typhimurium* growth was inhibited in various degrees of efficacy^28^. NPs are particulate dispersions or solid particles with a size range of 10–1000 nm. A nanoparticle matrix is used to dissolve, entrap, encapsulate, or bind the medication. One can obtain nanoparticles, nanospheres, or nanocapsules depending on the preparation technique used^29^. Nanometals were distinguished by their oligodynamic impact, which was the microbicidal action of metals, particularly heavy metals, at low concentrations^30^. Copper oxide nanoparticles (CuO NPs) are extremely reactive because of their high surface-to-volume ratio, which enhances their antimicrobial activities^31,32^. CuO NPs provide distinctive characteristics that have made them valuable for a variety of applications, including sensors and extremely strong materials^33^, and have strong antibacterial activity^34^. It is commonly known that both Gram-positive and Gram-negative bacteria can be killed by the surface of objects made of copper^35^. CuO NP have been emphasized as a bactericidal agent against a variety of bacteria, including film-forming bacteria like *Klebsiella pneumonia* and *Enterococcus faecalis* bacteria linked to nosocomial infections, as well as methicillin-resistant *Staphylococcus aureus* (MRSA)^36^. The primary mechanisms responsible for the antibacterial action of copper are attributed to the generation of reactive oxygen species (ROS) and the release of ions (Cu + and Cu^2^+)^37^. In this study, *Simmondsia chinensis* (Joboba) and *Azadirachta indica* (Neem) plants were collected for the biogenic synthesis of CuO NPs because they contain bioactive secondary metabolites, which include phenols, terpenes, and alkaloids and provide protection from microorganisms and herbivores^38,39^. Our hypothesis to test Neem and Jojoba extracts for the synthesis of CuO NPs with antibacterial activity. In this study, we analyzed CuO NPs using different methods, including UV‒Vis spectroscopy, FTIR spectroscopy, SEM-EDX, and TEM. We also investigated the effect of CuO NPs against multidrug-resistant pathogens, focusing on the antibacterial, antibiofilm, antioxidant, and cytotoxic properties of the CuO NPs, as shown in Fig. 1.

## Results

### Clinical isolates

**Fig. 2:**
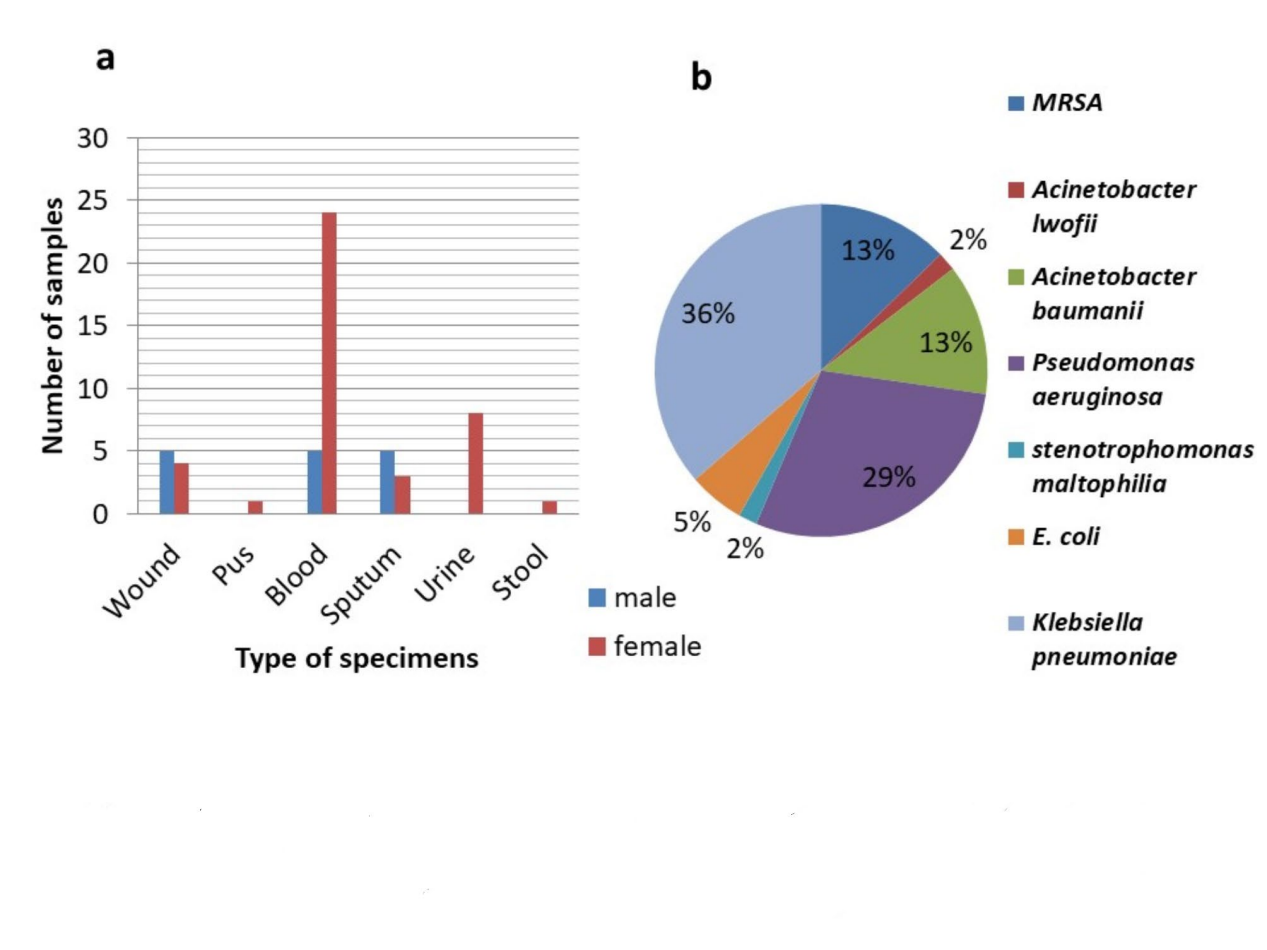
(**a**) Distribution of bacterial isolates obtained from patients of different sexes and (**b**) percentages of the pathogenic bacterial isolates causing healthcare-associated infections isolated in the present study.

The isolates were identified by using the vitek-2 apparatus, as shown in Fig. 2. The clinical isolates obtained were identified as follows: *E. coli* (E1 to E3), *Klebsiella pneumoniae* (K1 to K20), *Acinetobacter* spp. (A1 to A8), *Pseudomonas aeruginosa* (P1 to P16), MRSA (M1 to M7), and *Stentotrophomonas maltophilia* (S1). The most prevalent isolate obtained from the hospital was *Klebsiella pneumoniae* (38%), followed by *Pseudomonas aeruginosa* (29%). The lowest number of isolates obtained belonged to *Acinetobacter* spp. and *Stentotrophomonas maltophilia*, with percentages of 15% and 2%, respectively, as shown in Fig. 2b. Fifty-five bacterial pathogens were obtained from different patients from different specimens, as shown in Fig. 2a. The most common isolates were isolated from blood specimens (52.7%), followed by those from wound specimens (16.3%).

### Biofilm production

All the bacterial isolates were tested for their ability to produce biofilms. All the isolates of *E. coli* were strong biofilm producers; on the other hand, all the isolates of *Stenotrophomonas maltophilia* and *Pseudomonas aeruginosa* were nonbiofilm producers, as shown in Fig. 1 in the supplementary file. All the isolates of *Klebsiella pneumoniae* and MRSA produced biofilms to different degrees.

### Characterization of the biosynthesized CuO NPs and ethanolic plant extracts

#### TEM analyses

**Fig. 3:**
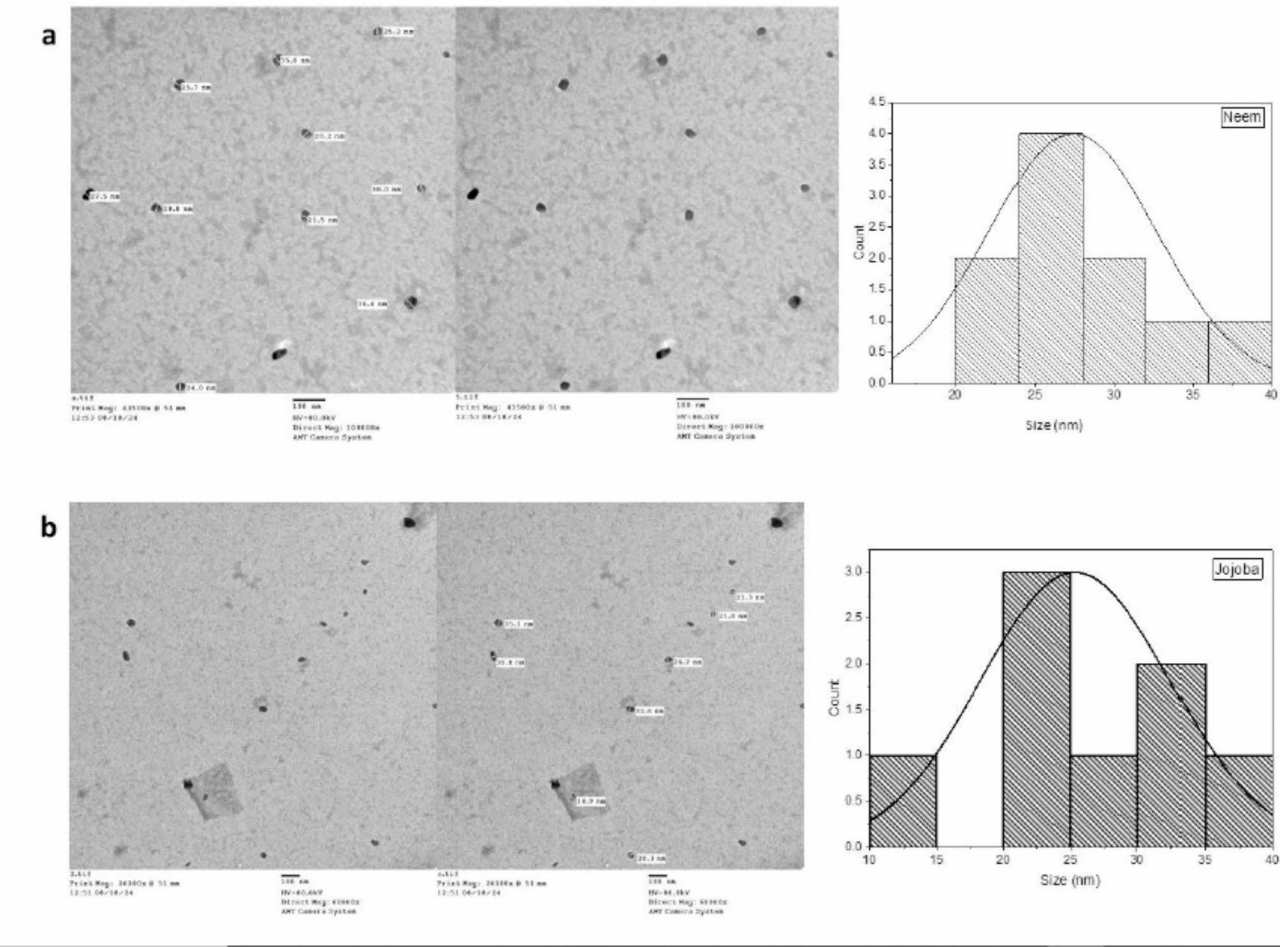
TEM images at 20000x, and histogram distribution plot of (**a**) CuO NPs biosynthesized by ethanolic Neem extract and (**b**) CuO NPs biosynthesized by ethanolic Jojoba extract.

The biosynthesized nanoparticles can be observed in a typical transmission electron microscope image, as shown in Fig. 3. TEM micrographs CuO NPs, revealed that the CuO NPs were semispherical in shape. The homogeneous distribution of CuO NPs and the diameter of the particles from various grid locations were also measured to determine the particle size distribution, with an average particle size ranging from 30.9 nm to 10.7 nm.

### FTIR analyses of biosynthesized CuO NP and ethanolic plant extract

**Fig. 4:**
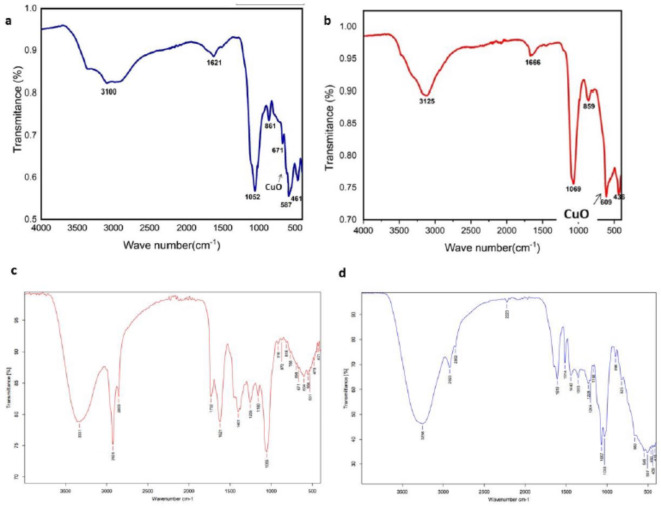
FTIR spectrum of (**a**) CuO NPs biosynthesized by ethanolic Neem extract (**b**) CuO NPs biosynthesized by ethanolic Jojoba extract (**c**) ethanolic Neem extract, and (**d**) ethanolic Jojoba extract.

**Fig. 5:**
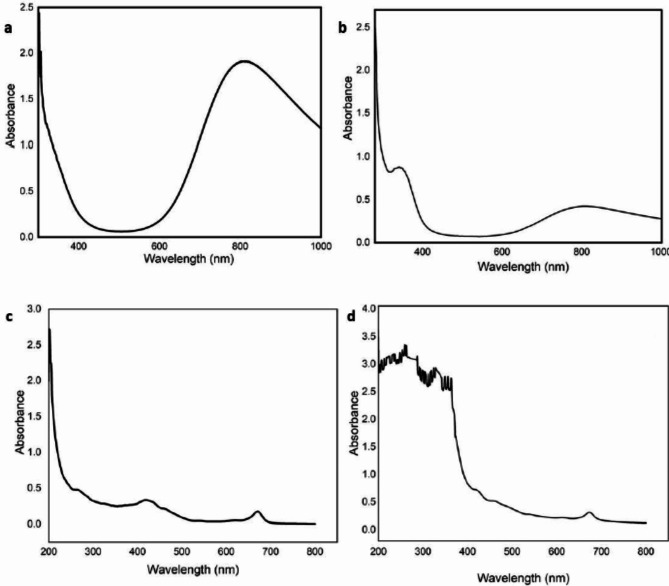
UV‒Vis absorption spectrum of (**a**) CuO NPs biosynthesized by ethanolic Neem extract (**b**) CuO NPs biosynthesized by ethanolic Jojoba extract (**c**) ethanolic Neem extract, and (**d**) ethanolic Jojoba extract.

**Fig. 6:**
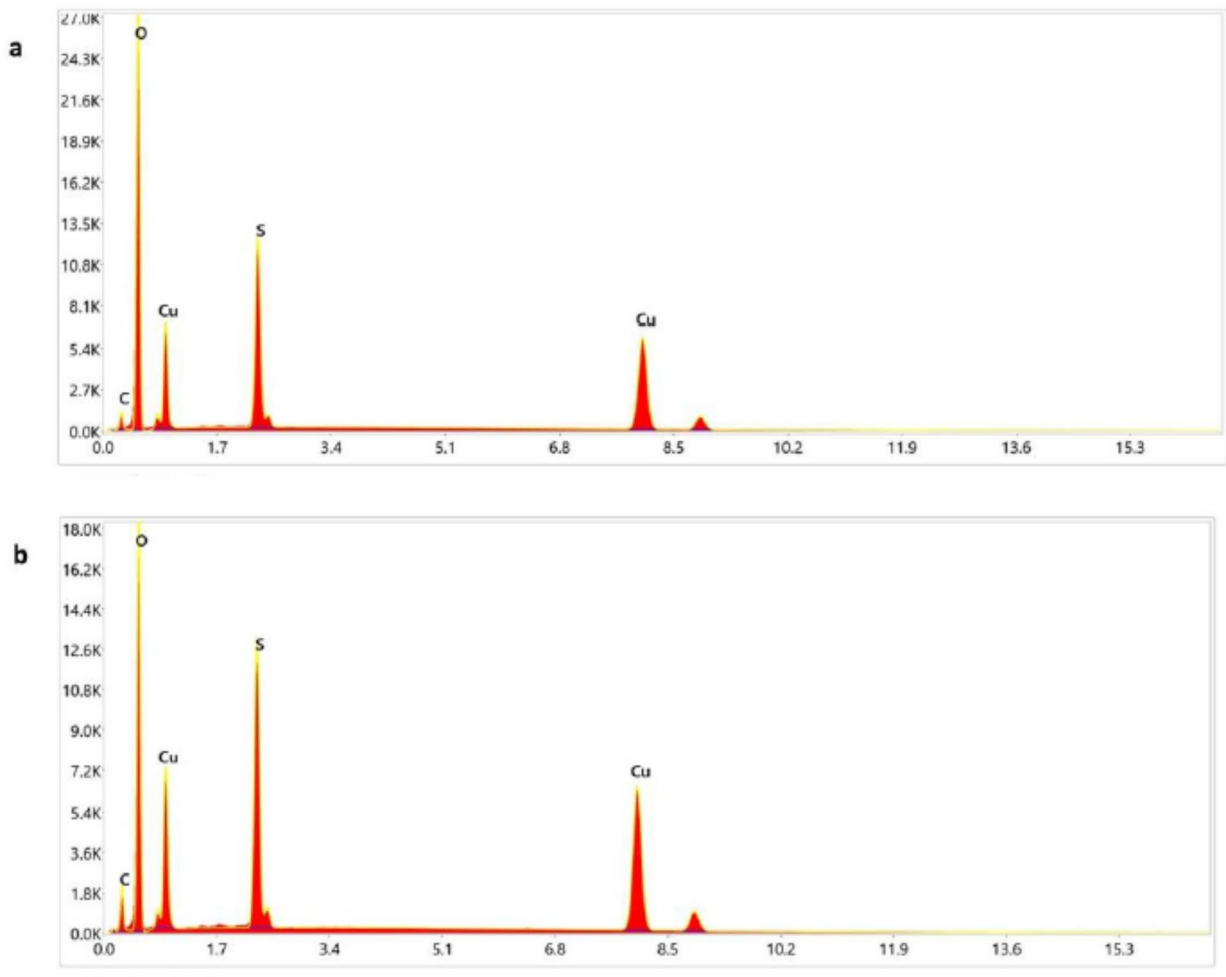
EDX analysis of CuO NP biosynthesized by (**a**) ethanolic Neem extract, abd (**b**) ethanolic Jojoba extract.

FTIR analysis was performed on the biosynthesized CuO NPs to investigate the functional groups on the surface of the bio-CuO NPs. The peaks at ~ 611 cm − 1 and ~ 463 cm − 1 corresponded to Cu₂O and CuO, respectively. The intense bands at 1669 and 1627 cm − 1 are attributed to the carbonyl group involved in the creation of the NPs. Cu-O-Cu is represented by a broad band between 400 and 800 cm-1. Moreover, the two bands of OH groups appear at ~ 3128 and ~ 1669 cm-1. The vibrations of the C–H groups were attributed to stretching and bending at ~ 2498 cm-1. At 1449–1669 cm-1, the aromatic C = C groups also exhibited bending (Figs. 4a and b, 5). In addition, FTIR measurements were performed to determine the biomolecules in the plant extracts. As shown in Fig. 6, prominent IR bands are observed at 3331, 2926, 2855, 1732, 1621, 1442, 1353, 1067, 872, 768 and 463 cm^− 1^. The bands located at approximately 3,300, 2,900, 1,600, 1,400, and 1,020 cm^− 1^ are attributed to the functional groups found in the specimen. Additionally, they match OH bonding, indicating the presence of phenol, CH alkyl, C = N, NH, and CO stretching, in that order. The majority of the IR bands correspond to the flavonoids and tepenoids that are present in the neem and jojoba extracts. The sharp bands at 2926 and 2855 cm^− 1^ originated from C–H stretching modes. The stretching vibrations of C = O and C = C are attributed to the medium-intensity bands at 1732 and 1621 cm^− 1^, respectively. The –C–O and –C–O–C stretching modes can be responsible for the absorption bands at 1353 and 1067 cm^− 1^. The aromatic amine group’s C–N stretching mode gives rise to the moderately intense band at 1442 cm^− 1^. The vibrational bands corresponding to bonds such as –C = C, –C = O, –C–O, –C–O–C and –C–N are derived from water soluble compounds such as flavonoids, terpenoids and thiamine present in neem and jojoba (Fig. 4c and d).

### UV‒visible spectroscopy

The UV–Vis spectroscopy analysis of both ethanolic plant extracts and biosynthesized CuO NPs were characterized in a comparison as shown in Fig. 5. The Uv–Vis spectrum of ethanolic Neem extract at 265 nm, 422 nm and 670 nm for ethanolic Neem extract while, Jojoba extracts detected at 265.5 nm, On other hand, CuO NP biosynthesized by ethanolic Jojoba and Neem extracts surface plasmon band was detected at 344–345 nm, respectively.

### EDX analyses

A strong peak corresponding to the Cu atom was recorded alongside weak signals from the C and O atoms. The EDX analysis showed that C and O were present, although C was present in tiny amounts. Consequently, the study showed that the obtained copper oxide nanoparticles were extremely pure (Fig. 6).

### Antibacterial activity of CuO NPs

**Fig. 7:**
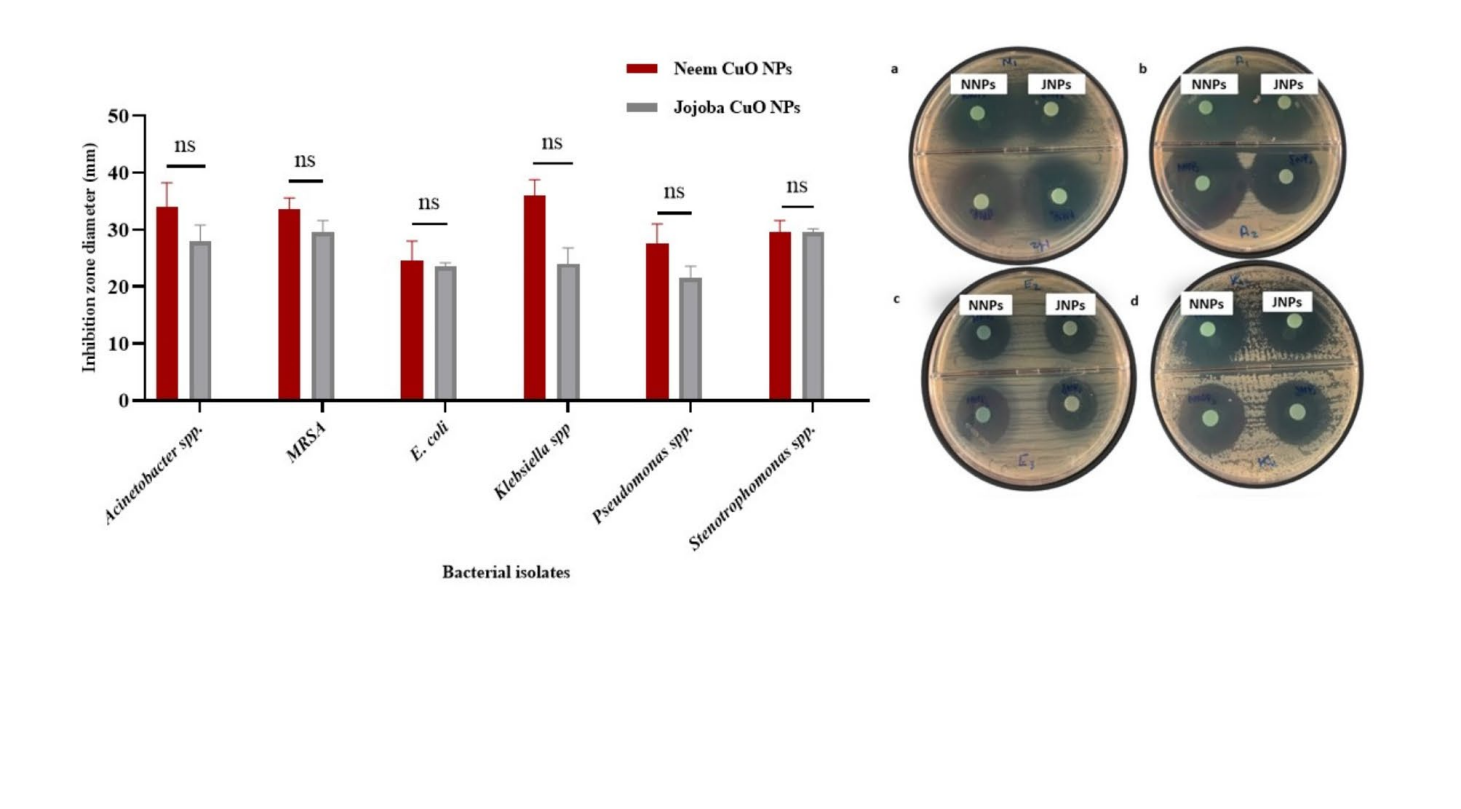
Screening of the antibacterial activity of the biosynthesized CuO NPs against (**a**) MRSA, (**b**) *Acinetobacter* spp., (**c**) *E. coli*, and (**d**) *K. pneumoniae*. The antibacterial activity was evaluated by the agar diffusion method. All the nutrient agar plates were inoculated in duplicate with 100 µl of each freshly tested culture with a sterile cotton swab, and the paper disk was immersed in each sample of biosynthesized CuO NPs for 10 s and subsequently placed on inoculated plates. The plates were incubated at 37 °C for 24 h (P value = 0.0594; ns; no significant difference).

CuO NPs biosynthesized by the ethanolic Neem extract showed potent antibacterial activity against the tested isolates. The results showed that the inhibition zone diameter of biogenic CuO NPs by ethanolic Neem extract ranged from 35 to 24 mm, while the diameter of the zone inhibited by the ethanolic Jojoba extracts ranged from 30 to 19 mm, as shown in Fig. 7 and supplementary Table 1.

### Determination of the MIC of biosynthesized CuO NPs

Biogenic CuO NPs had significant antibacterial effects on all the pathogens in a dose-dependent manner. As shown in Table 1. The findings indicated that the MIC of CuO NPs biosynthesized by the ethanolic Neem extract was 125 µg/mL for MRSA, *Acinetobacter* spp., *K. pneumoniae* and *P. aeruginosa* and 62.5 µg/mL for *Stenotrophomonas* spp. and *E. coli.* The MIC of CuO NPs biosynthesized by the ethanolic extract of Jojoba was 125 µg/mL for all pathogens except *Stenotrophomonas* spp. (62.5 µg/mL). The MBC/MIC ratio for all the tested strains showed that the tolerance level of CuO NPs was one, which indicates that the biosynthesized CuO NPs are considered bactericidal agents (Fig. 2 in the supplementary file).

**Table 1:** MIC and MBC of the biosynthesized CuO-NPs.

Isolate	CuO-NPs biosynthesized by ethanolic neem extract (µg/ml)			CuO-NPs biosynthesized by ethanolic jojoba extract (µg/ml)		
	MIC	MBC	MBC/MIC	MIC	MBC	MBC/MIC
*E. coli*	62.5	62.5	1	125	125	1
*Klebsiella pneumonia*	125	125	1	125	125	1
*Pseudomonas aeruginosa*	125	125	1	125	125	1
*Acinetobacter* spp.	125	125	1	125	125	1
*Stenotrophomonas maltophilia*	62.5	62.5	1	62.5	62.5	1
MRSA	125	125	1	125	125	1

### Antibiofilm of CuO NPs against the tested strains

**Fig. 8:**
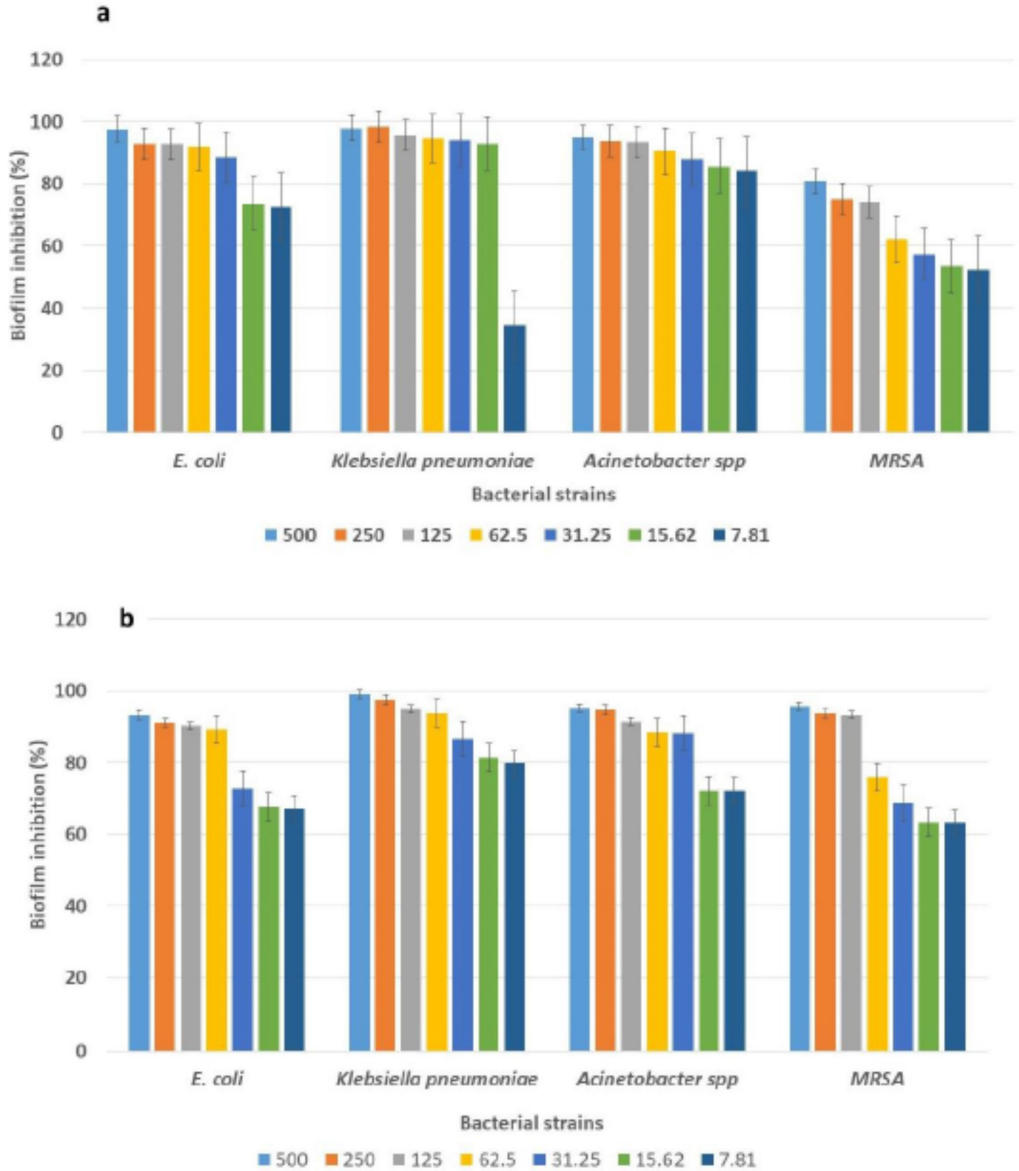
Antibiofilm activity of CuO NPs biosynthesized by (**a**) ethanolic Neem extract and (**b**) ethanolic Jojoba extract, expressed as a percentage of the total inhibition. The 96-well polystyrene microtiter plates were inoculated in duplicate with 100 µL of tryptone soy broth, and 100 µL of diluted biosynthesized CuO NPs was incubated at 37 °C for 24 h. Then, 200 µL of a 0.1% crystal violet solution was added to the wells for 15 min. After washing three times and drying, 200 µL of 30% glacial acetic acid was added to the wells, and the absorbance was subsequently measured at 570 nm.

The findings showed that, in a concentration-dependent way, the treated isolates significantly reduced the production of biofilm compared to the untreated cells. According to the CV assay, CuO NPs biosynthesized by the ethanolic Neem extract decreased the initial bacterial adhesion by 74.2–92.9% and 62.2–92%, respectively, at 125 –62.5 µg/ml. On the other hand, CuO NPs biosynthesized by the ethanolic extract of Jojoba reduced the initial bacterial adhesion by 90.2–95% and 87.2–93.7% at 125 –62.5 µg/ml, respectively (Fig. 8 and supplementary Table 2, Table 2).

### Antioxidant activity of the biosynthesized CuO NPs

**Fig. 9:**
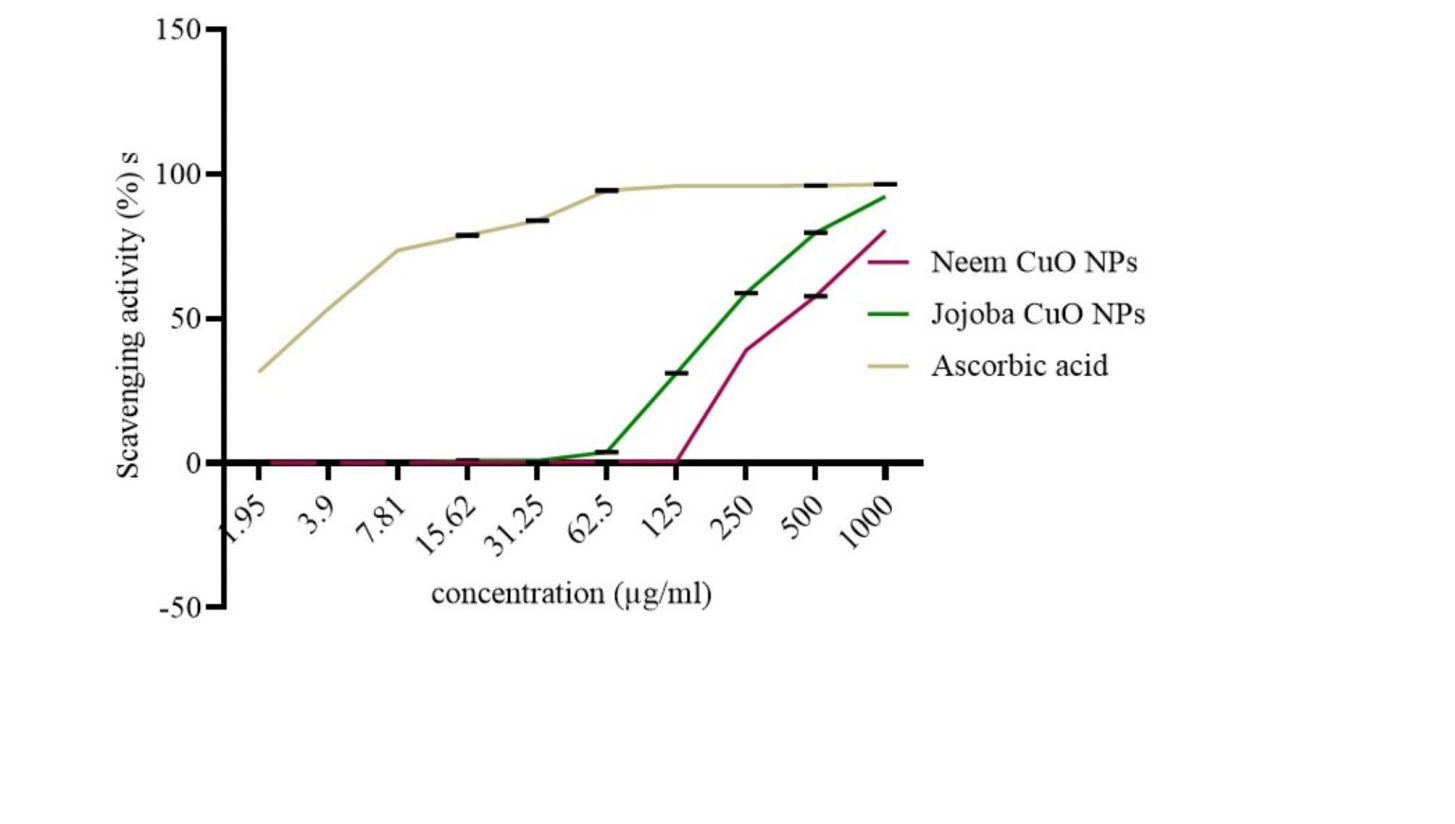
Antioxidant activity of the biosynthesized CuO NPs. Using the 2,2 DPPH assay, expressed as DPPH scavenging %, ascorbic acid was used as a control.

The antioxidant activity of CuO NPs was evaluated at different doses (1000, 500, 250, 125, 62.5, 31.25, 15.62, 7.81, and 3.9 µg/ml) by the DPPH free radical scavenging test, using ascorbic acid as a positive control. The average percentage inhibition values of the biosynthesized CuO NPs by Neem and Jojoba were (39, 57.7, and 80.5%), (31.1, 58.8, 79.6, and 92.2%), respectively. As the NP concentration increased, the ascorbic acid concentrations were 95.11, 95.25, and 95.75%, respectively (Fig. 9).

### Cytotoxicity of the biosynthesized CuO NPs against the HFB4 cell line

**Fig. 10:**
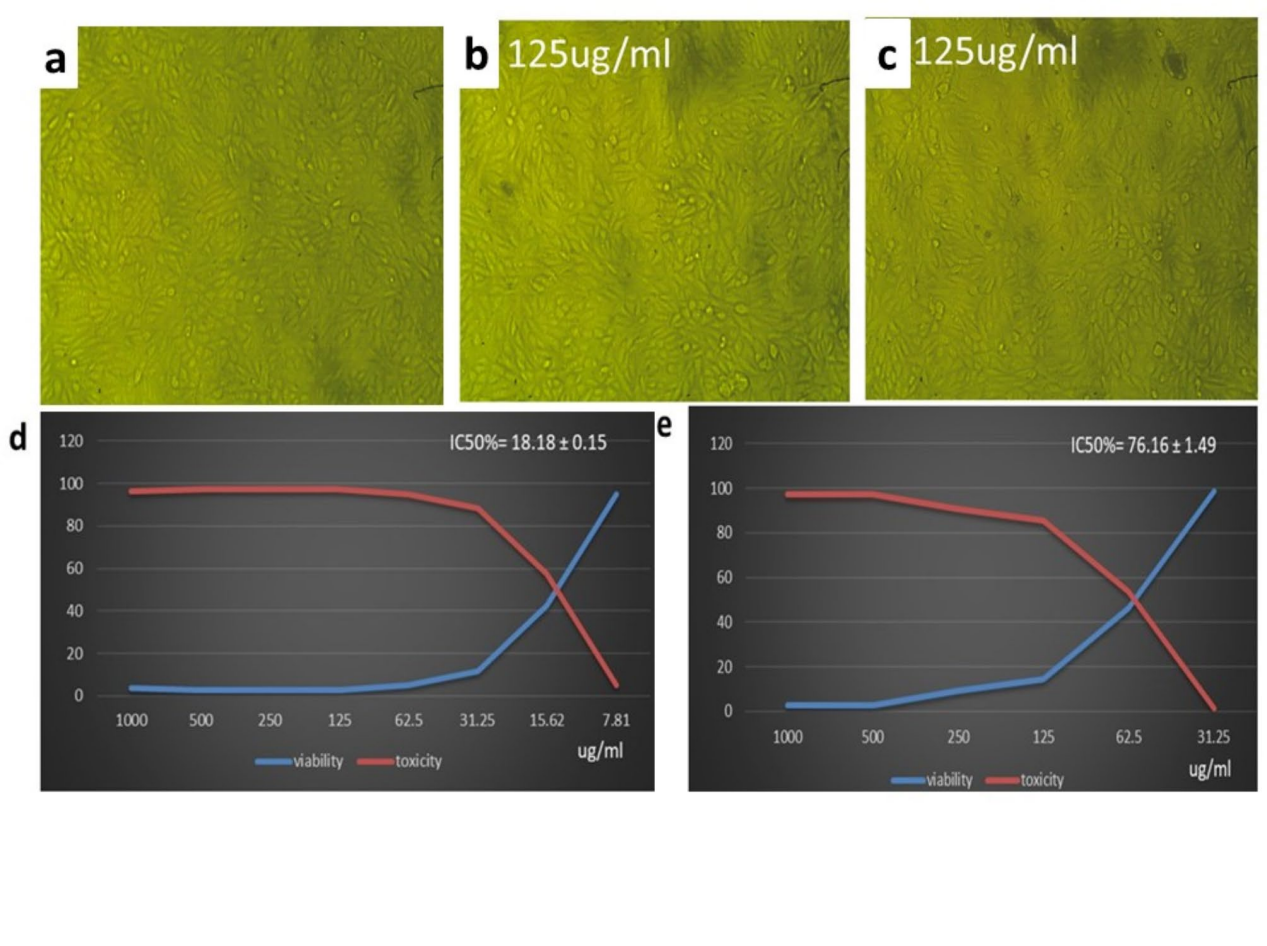
Effect of CuO NPs biosynthesized by ethanolic extracts of (**b**,**d**) Neem and (**c**,**e**) Jojoba on HBF4 cells at different concentrations, while (**a**) control cells.

The first and most obvious observation that occurred after exposure to CuO NPs was a change in cell shape and morphology. As a result, a light inverted microscope may be used to observe the damage that the CuO NP exposure dosage causes to cellular morphology and shape. The MTT assay is a sensitive colorimetric method used in cell proliferation and cytotoxicity tests to determine the number of viable cells. The death of normal cells subjected to varying doses of CuO NPs was also dose dependent, as demonstrated in Tables 3 and 4 in the supplementary file and Fig. 10. In particular, the IC50 for normal cells detected by HBF4 is shown in Fig. 10d and e. Therefore, it is highly recommended that treatments with CuO NPs be administered at concentrations less than 383.41 µg/ml to protect human safety.

### Bactericidal mechanism of biosynthesized CuO NPs

**Fig. 11:**
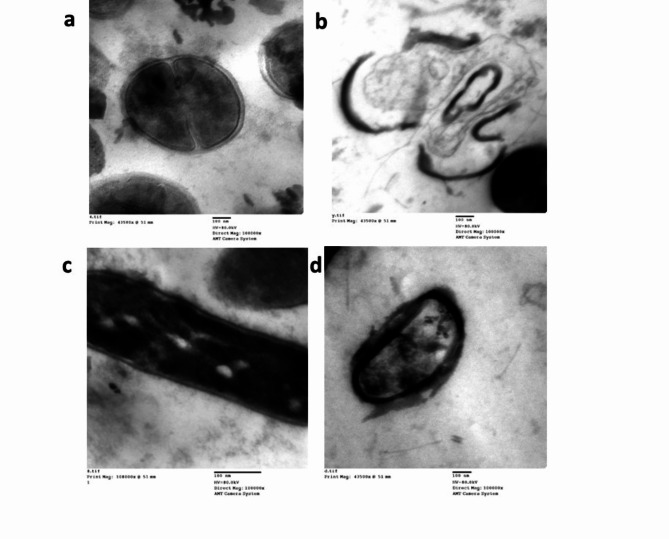
TEM images of (**a**) control MRSA, (**b**) treated MRSA with biogenic CuNPs, (**c**) untreated *K. pneumoniae*, and (**d**) *K. pneumoniae* treated with biogenic CuO NPs at 1000x. Stained cells were used as a negative control, while cells treated with CuO NPs were used as a positive control. Ultrastructural damage and deformation of the bacterial cell wall were induced by the MIC of CuO NPs after 1 h of exposure at 37 °C, while the untreated cells exhibited an intact bacterial membrane.

TEM analysis was carried out to observe the changes in bacterial cell morphology induced by the biosynthesized CuO NPs. *Klebsiella pneumoniae* and MRSA treated with PBs and 70% alcohol indicate live cells (control). Untreated MRSA cells exhibited a smooth and round morphology, as shown in Fig. 11a, while untreated *Klebsiella pneumoniae* cells exhibited an intact rod-shaped morphology, and the cell walls were rigid (Fig. 11c).

Treatment of *Klebsiella pneumoniae* cells with CuO NPs led to shrinkage and wrinkling of the cell surface, which are characteristic of morphological damage (Fig. 11d). TEM images of MRSA revealed a condensed intracellular matrix as a result of CuO NP treatment and fragmentation of the cytoplasmic membrane, which led to leakage of cytoplasmic cellular components (Fig. 11b).

### Molecular docking

Molecular docking studies of CuO-NPs against crystal structures of penicillin-binding protein 4 (PBP4) and OXA-48 beta-lactamase (PDB ID: 1TVF and 7AUX, respectively) have been performed as further evidence for biological screening investigations. The data are shown in Tables 2 and 3, revealing high agreement between the docking and experimental results.

CuO-NPs exhibited good in silico inhibition of both proteins 1TVF and 7AUX, with binding free energies of -4.19016 kcal/mol and − 3.07936 kcal/mol, respectively, as demonstrated by their favorable interactions with the chosen residues, low RMSD values, and low docking scores. A low binding affinity indicates that the energy required for drug-receptor interactions is minimal, resulting in a more durable interaction and increased activity of the OXA-48 beta-lactamase (7AUX) bound to CuO-NPs through amino acids.

**Fig. 12:**
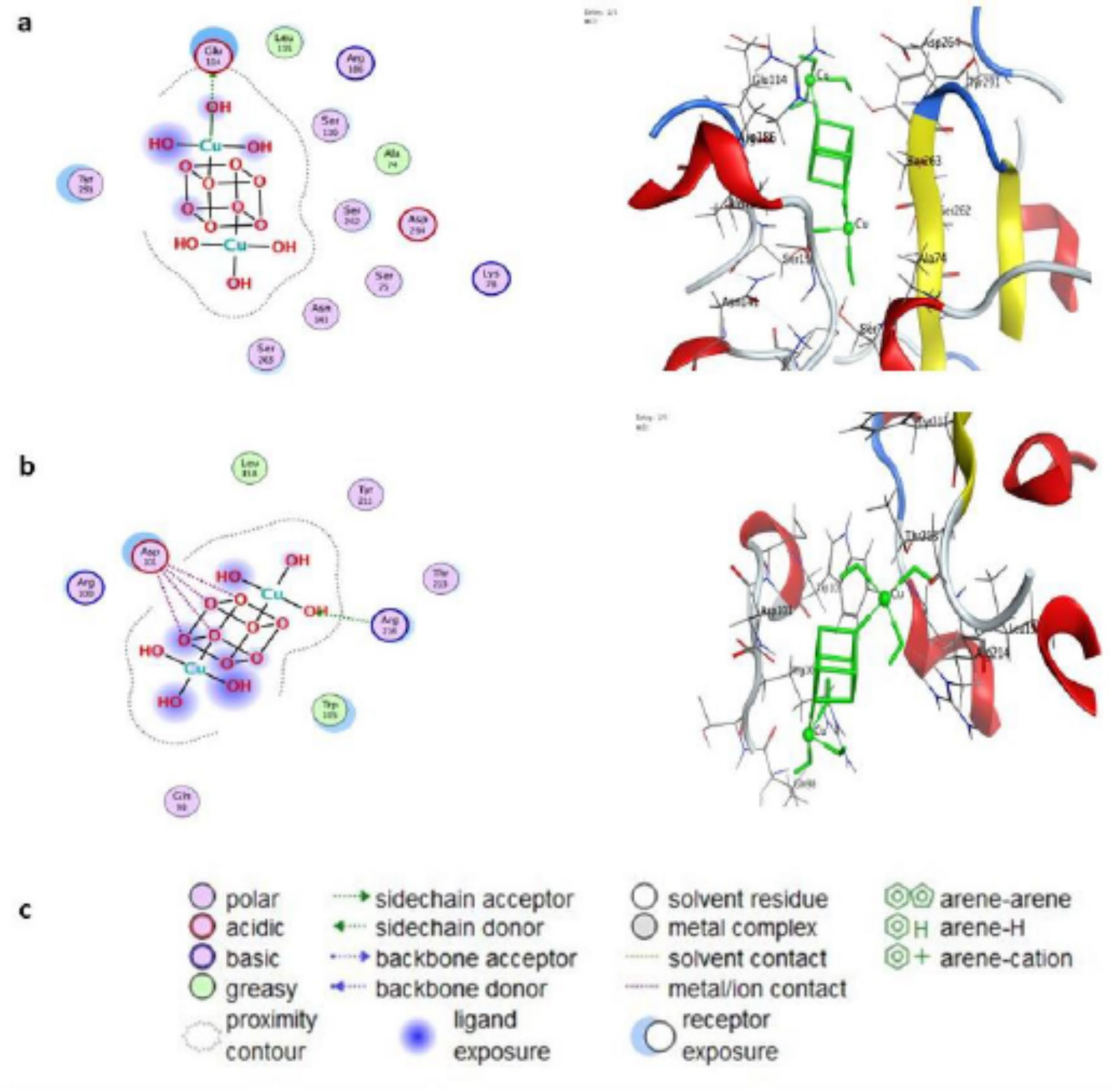
Molecular docking analysis of 2D and 3D images showing (**a**) the interaction between CuO NPs and active sites of the 1TVF protein, (**b**) the interaction between CuO NPs and active sites of the 7AUX protein, and (**c**) the key factors involved in the interactions between CuO NPs and protein receptors.

The best-fit 2D and 3D poses adopted by CuO-NPs are displayed in Fig. 12, and the binding energy scores are presented in Table 2. CuO-NPs formed one donor hydrogen bond with the penicillin-binding protein (1TVF) residue and GLU 114 via 9 Cu atoms. On the other hand, H-acceptor interactions were found between the O14 atom of the ligand and the ARG214 residue of the 7AUX enzyme. Table 3 lists the hydrogen bonds that exist between the identified proteins and the studied NPs. The binding proteins interact with the investigated compound by stabilizing them in the receptor’s amino acid cavity, inhibiting the growth of the targeted proteins.

**Table 2:** Docking scores and energies of CuO with the crystal structure of (PBP4) from *Staphylococcus aureus* (PDB ID: 1TVF) and OXA-48 beta-lactamase from *K. pneumoniae* (PDB ID: 7AUX) receptors.

Mol	Protein	S	rmsd_refine	E_conf	E_place	E_score1	E_refine	E_score2
CuO-NP	1TVF	-4.19016	6.479323	− 1422.3	− 85.3459	− 17.6603	− 23.2547	− 4.19016
CuO-NP	7AUX	− 3.07936	2.927132	− 1412.15	− 63.1728	− 11.951	− 6.38765	− 3.07936

**Table 3:** Interaction of CuO with the crystal structure of PBP4 from *Staphylococcus aureus* (PDB ID: 1TVF) and OXA-48 beta-lactamase from *K. pneumoniae* (PDB ID: 7AUX) receptors.

Mol	Protein	Ligand	Receptor	Interaction	Distance	E (kcal/mol)
CuO-NP	1TVF	CU 9	OE1 GLU 114 (A)	H-donor	2.78	− 0.5
CuO-NP	7AUX	O 14	NE ARG 214 (A)	H-acceptor	2.93	− 0.5
		O 2	OD2 ASP 101 (A)	Ionic	2.80	− 6.0
		O 3	OD2 ASP 101 (A)	Ionic	2.80	− 5.9
		O 6	OD2 ASP 101 (A)	Ionic	2.67	− 7.1
		O 7	OD2 ASP 101 (A)	Ionic	2.70	− 6.8

## Discussion

Biological synthesis of nanoparticles utilizing plants as bioreductants can be beneficial over other biological processes since the process can be scaled up appropriately for large-scale synthesis and does not require the maintenance of cell culture. The method used to synthesize nanoparticles can impact their stability. Green synthesis methods, which use plant extracts or microorganisms, often result in nanoparticles with good stability due to the presence of natural capping agents. The shelf-life of biosynthesized copper oxide nanoparticles (CuO NPs) can vary depending on several factors, including the synthesis method and storage conditions. Generally, biosynthesized CuO NPs are known for their stability. For instance, CuO NPs synthesized using plant extracts have shown stability for over 30 days due to the protective capping agents from the biomolecules^40^. In this study, ethanolic extracts of *Azadirachta indica* and *Simmondsia chinensis* were used as reactants to nucleate nanoparticles in solution. The synthesis of CuO NPs was confirmed by the change in the color of the reaction mixture and by UV‒visible spectroscopy. When plant extracts were added to the CuSO4 solution, CuO NP production was indicated by the change in color from pale blue to green to dark brown. The obtained results are intriguing since they can be used as a basis for identifying possible therapeutic plants for synthesizing CuO NPs. *A. indica* extract contains biomolecules that can reduce metal ions to metal NPs, including terpenoids, nimbaflavone, and polyphenols^41–43^. Our results are unique since this is the first study to mention the biosynthesis of CuO NPs by using Neem and Jojoba extracts to overcome multidrug resistance in Egypt.

The biosynthesis of CuO NPs was characterized by several techniques. The UV‒Vis spectroscopy results of the CuO NPs showed an absorption peak at 344–345 nm, which confirmed the formation of CuO NPs. According to the authors, the presence of copper oxide is indicated by an absorbance peak at 386 nm in the UV‒vis spectrum^44^. The FTIR results for the synthesized copper oxide nanoparticles are shown in Fig. 4a and b. The size, morphology, and form of the bio-CuO NPs were determined via transmission electron microscopy (TEM). Figure 4 showed the size distribution and shape of biosynthesized CuO NPs. TEM images confirmed the formation of nanocrystalline CuO Nps with an average size of 25.3, and 27.4 nm fou CuO NPs biosynthesized by Jojoba and Neem, respectively. In accordance with^45^, CuO NPs with a spherical shape were observed.

FTIR spectroscopy is an efficient way to investigate the potential interactions of CuO NPs with different functional groups^46^. Other functional groups appear at various locations in the FTIR spectrum (Figs. 4d and 6d). FTIR spectrum reported that Neem and Jojoba extract contain several bioactive compound such as alkaloids, steroids, flavonoids, and tannins which reduce and stabilize the synthesized CuO NPs^47^. They enhance stability of NPs and also prevent their interaction with atmospheric oxygen. These NMs are thus not oxidized and can be kept for long period of time without undergoing any change in their properties^48^. The carbonyl group that forms nanoparticles is represented by the powerful bands at 1627 and 1669 cm − 1. Both the -OH groups in alcohols and the -NH2 groups in primary aromatic amines are responsible for another strong, intense band at 3128 cm − 1^49^. Similarly, as indicated in Fig. 4aand 4b^50^, the presence of bands at 611 cm − 1 in the FTIR spectra of the biogenic CuNPs might be attributed to the occurrence of CuO. The EDX profile of the CuNPs demonstrated substantial elemental signals corresponding to copper, confirming the presence of CuO NPs. The carbon and oxygen found in organic molecules that surround the nanoparticles, such as capping molecules and the tape used to mount the CuO NPs, make up the remaining weight%^51^. In the line of our study, Aaga, and Anshebo^52^, XRD analysis of biosynthesized CuO NP showed that the CuO NPs were crystalline.

In vitro, the antimicrobial activity of the biosynthesized CuO NPs was evaluated against G + ve (MRSA) and G-ve (*E. coli*,* Klebsiella pneumoniae*,* Pseudomonas aeruginosa*,* Acinetobacter* spp., and *Stenotrophomonas maltophilia*) bacteria using the agar diffusion method. We focused on Gram-negative bacteria due to its outer membrane which makes Gram-negative bacteria more resistant to antibiotics than Gram-positive ones. In addition, Gram-negative bacteria can cause serious diseases in humans, especially in immuno-compromised individuals. Nosocomial infections caused by Gram-negative bacilli (GNB) are the most challenging issue for healthcare professionals due to antibiotic resistance^53^. The inhibition zone differed between Gram-negative bacteria (19 to 34 mm) and Gram-positive bacteria (28 to 32 mm). CuO NPs were effective against *Acinetobacter* spp. followed by *Stenotrophomonas maltophilia*, this disagree with Yassin et al.^54^, who reported that *Acinetobacter* spp. was resistant to AgNPs. The most resistant bacteria to CuO NPs were *E. coli* and *Pseudomonas aeruginosa*. This difference is mainly due to the differences in the structure of the bacterial membranes. This finding disagrees with Priyadarshini et al.^55^, who reported that *E. coli* was sensitive to TiO2NPs. According to a previous study, nanoparticles inhibit the growth of bacteria by interacting with the phosphorous moiety in DNA. This inactivates DNA replication and consequently lowers the activity of enzymes^56–58^. Additionally, it can inhibit respiratory enzymes in bacteria and halt the synthesis of ATP, which kills cells. Additionally, several changes, including membrane detachment, cytoplasmic shrinkage, and eventually membrane rupture, are caused by the electrostatic interactions between the positive charge of the NPs and the negative charge of the bacterial surface^59,60^. The total MICs for various extracts that resemble CuO NPs confirm that the addition of phytochemicals to various nanoformulations increases their activity. The results showed that even at low concentrations, the CuO NPs biosynthesized from the Neem and Jojoba ethanolic extracts prevented the growth of the tested bacteria. The MIC of CuO NPs biosynthesized by the ethanolic Neem and Jojoba extracts ranged from 62.5 to 125 µg/ml. These findings are consistent with those of Marzban et al.^61^, who reported that the MICs of biosynthesized CuO NPs ranged from 150 to 250 µg/ml.

**Fig. 13:**
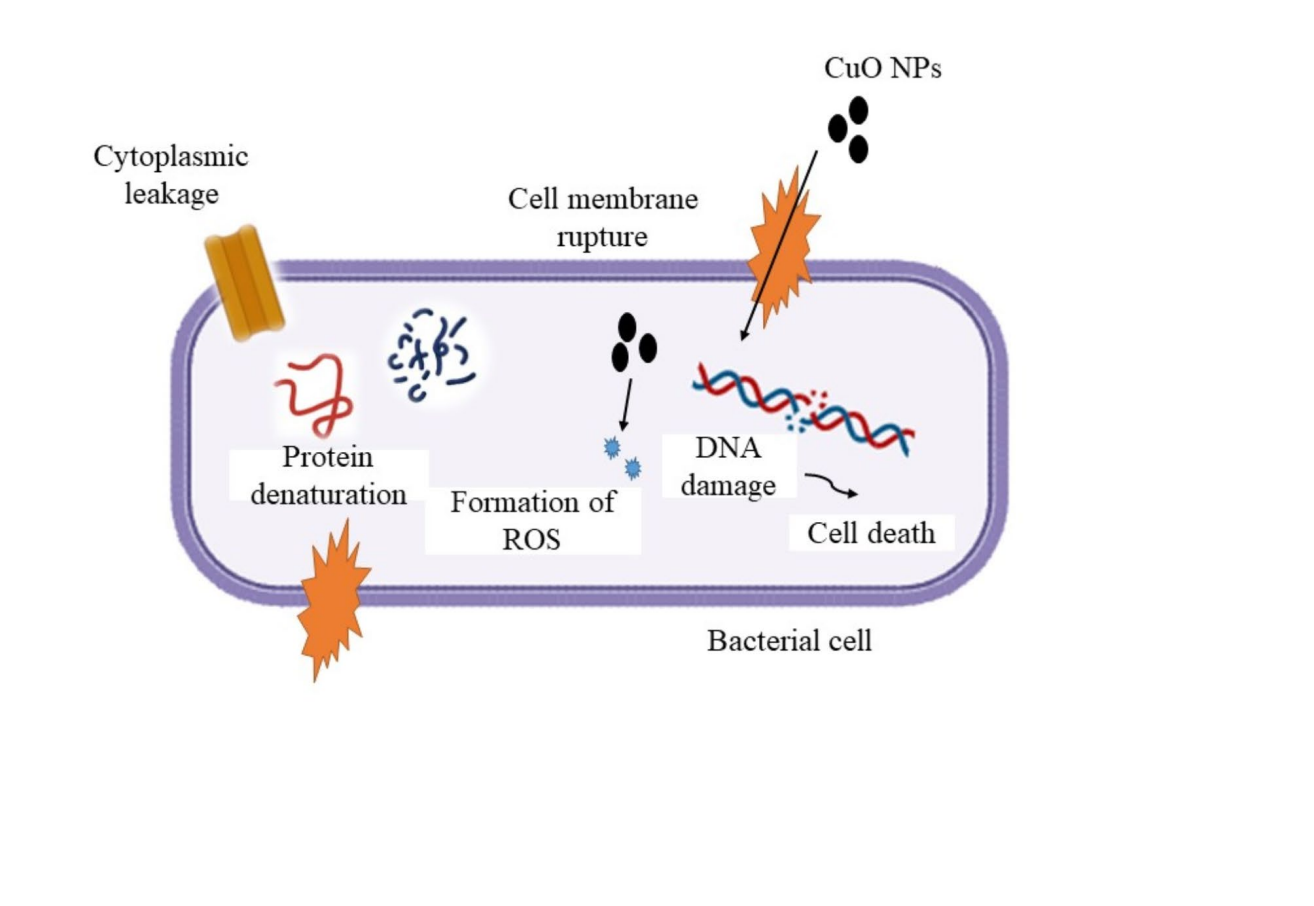
Antibacterial mechanism of CuO NPs (ROS: reactive oxygen species).

According to Tomayo et al.^62^, the bacteriolytic mode of copper ions involves switching the semipermeable membrane properties, which prevents bacterial cells from controlling transport through the plasma membrane and results in cell lysis and the release of cytoplasmic materials outside the membrane, as shown in Fig. 13. CuO NPs were found in an aggregated form, as demonstrated by the TEM images (Fig. 11b and d). Bacteria have an overall negative charge at biological pH levels due to the excess of carboxylic groups in the lipoproteins at the bacterial surface, which dissociate to leave the cell surface negative^63^. Because of electrostatic interactions, it is believed that the opposing charges of the bacteria and the copper ions produced by the nanoparticles cause adherence and bioactivity. Peptidoglycans bind Cu2 + ions produced from copper nanoparticles in liquid growth media because they are negatively charged molecules^64^. TEM micrographs, however, revealed that there was no direct contact between the different particles, which may be explained by the capping molecules that are present stabilizing the nanomaterial^65^. The bacterial ultrastructure of the control MRSA and *Klebsiella pneumoniae* cells were examined using TEM, which demonstrated that the cells were readable and that they did not appear to have been destroyed.

The antibiofilm activity of the CuO NPs had a range of effects on different bacterial isolates. The results showed that the CuO NPs biosynthesized by the ethanolic extract of Jojoba were better antibiofilm agents than were those synthesized by the ethanolic extract of Neem, and the inhibition percentages were 93.4 to 95% and 74.2 to 95.6%, respectively, at 125 µg/ml. Our results are similar to those of Agarwala et al.^66^, who showed that the antibiofilm effects of CuO NPs on MRSA and *E. coli* were 120 and 160 µg/ml, respectively. Several factors, such as the antimicrobial activity of the material, its biosorption-dependent mechanism, its size, its penetration capabilities, and other chemical properties influencing the ability of the material to form biofilms, could also account for the variation in inhibitory activity^67^.

Antioxidants combat reactive oxygen species (ROS), which are waste products of biological functions. Antioxidants can neutralize free radicals, which are responsible for several diseases^68^. The DPPH activity of nanoparticles increased in a dose-dependent way^69^. However, the CuO NPs biosynthesized by the Jojoba extract exhibited greater inhibition of DPPH scavenging activity than did the CuO NPs biosynthesized by the Neem extract. The antioxidant potential of CuO NP increased as the particle size decreased, supporting previous findings^70^.

The viability of normal HBF4 cells treated with the biosynthesized CuO NPs was assessed using the MTT assay. The toxicity of CuO NPs synthesized from ethanolic Neem and Jojoba extracts was tested in mammalian cells. The HBF4 cells treated with CuO NPs biosynthesized by Jojoba had significantly greater proliferation activity (IC50 value of 383.41 ± 3.4 µg/ml), whereas the IC50 value of CuO NPs biosynthesized by neem was 402.73 ± 1.86 µg/ml. Nearly all normal human HBF4 cells were shown to survive up to 250 µg/ml of biosynthesized CuO NPs. The CuO NPs biosynthesized by the ethanolic Neem and Jojoba extracts exhibited 99.7 and 99.2% cell viability at 125 µg/ml, respectively. Cytotoxicity was lower than that of Shiravand and Azarbani^71^, who reported that 88% of *Ferula macrocolea* flowers were biosynthesized at 100 µg/ml.

In silico, molecular docking is a type of bioinformatics modelling in which two or more molecules are combined to generate a stable adduct. they have been carried out on biosynthesized CuO NPs against PBP4 and OXA-48 beta-lactamase (PDB IDs: 1TVF and 7AUX, respectively). Molecular docking generates different possible adduct structures that are ranked and grouped using a scoring function in the software. The information obtained from the docking technique can be used to determine the binding energy, free energy, and stability of the ligand. The findings revealed that the biosynthesized CuO NPs effectively promoted the expression of essential bacterial proteins. The CuO NPs exhibited hydrogen bonds with the remaining material that had close interactions with the active sites^72^. The capacity of nanoparticles to interact with bacteria through electrostatic, van der Waals, or other hydrophobic interactions by stabilizing the binding proteins, inhibiting the growth of the targeted proteins, and determining their antibacterial efficacy^73,74^. These biological screening investigations have been supported by molecular docking research. Future work will be conducted based on testing shelf-life and the impact on environment and applications such as fighting of biofilm on heathcare settle surfaces.

All the results reported in the present study support the use of Neem and Jojoba extracts in place of hazardous chemical reductants to CuO NPs that can suppress the growth of various multidrug-resistant bacteria. Future research on copper oxide nanoparticles (CuO NPs) could indeed take several exciting directions such as combination with other antibacterial Agents, and Investigating the synergistic effects of CuO NPs when combined with other antibacterial agents could enhance their efficacy. This approach might help in overcoming bacterial resistance and improving the overall antibacterial activity.: Biofilms are complex communities of microorganisms that are notoriously difficult to eradicate. Studying the impact of CuO NPs on biofilms in real-world environments, such as medical devices or industrial pipelines, could provide valuable insights into their practical applications. Scaling Up Synthesis for Industrial Applications: Developing scalable and cost-effective synthesis methods for CuO NPs is crucial for their industrial application. This includes optimizing green synthesis techniques to produce large quantities of CuO NPs with consistent quality and minimal environmental impact.

## Materials and methods

During four months, from July 2023 to November 2023, fifty-five pathogenic isolates, including *E. coli*,* Klebsiella pneumoniae*,* Acinetobacter baumannii*,* Acinetobacter lwoffii*,* Pseudomonas aeruginosa*,* Stenotrophomonas maltophilia*, and methicillin-resistant *Staphylococcus aureus* (MRSA), were obtained from El-Galaa, El-Gawy, and Wadi El-Nile Hospitals through the Armed Forces laboratories for medical research and blood bank in Cairo, Egypt, and identified by a Vitek-2 apparatus (bioMérieux. Marcy l’Etoile, France) and the BioFire Film Array test. The isolates were collected from 31 females whose ages ranged from 13 to 86 years and 24 males whose ages ranged from 34 to 90 years.

### Standard inoculum

The density of the inocula was standardized to 10^6^ CFU/ml as follows: The tested organism was inoculated into 5 ml of nutrient broth and incubated for 24 h at 37 °C. Then, 0.2 ml from a 24 h culture was added to 20 ml of sterile nutrient broth and incubated for 3–4 h to produce a bacterial suspension with turbidity comparable to that of 0.5 McFarland solution^75^. The plates were inoculated within 15 min to avoid changes in inoculum density^76^.

### Detection of biofilm formation

We used the Congo red agar (CRA) method to test biofilm formation in several samples according to the guidelines of Freeman et al., 1989^77^. The bacterial isolates (as indicated in methodology Sect. 2) were cultured on Congo red agar plates and incubated at 37 °C for 24–48 h. The following three CRA strains were prepared: Nutient agar (28 g/L), sucrose (5%) and Congo red stain (0.8 g/L) (HiMedia, India). Congo red is a highly concentrated aqueous solution that is added after the agar has cooled to 55 °C and autoclaved (121 °C for 20 min) alone. Black colonies with a dry crystalline consistency were recorded as strong biofilm producers. Dark colonies without a dry crystalline shape were recorded as moderate biofilm producers. Colonies that remained pink were recorded as nonbiofilm producers^78^.

### Collection of plant materials

**Fig. 14:**
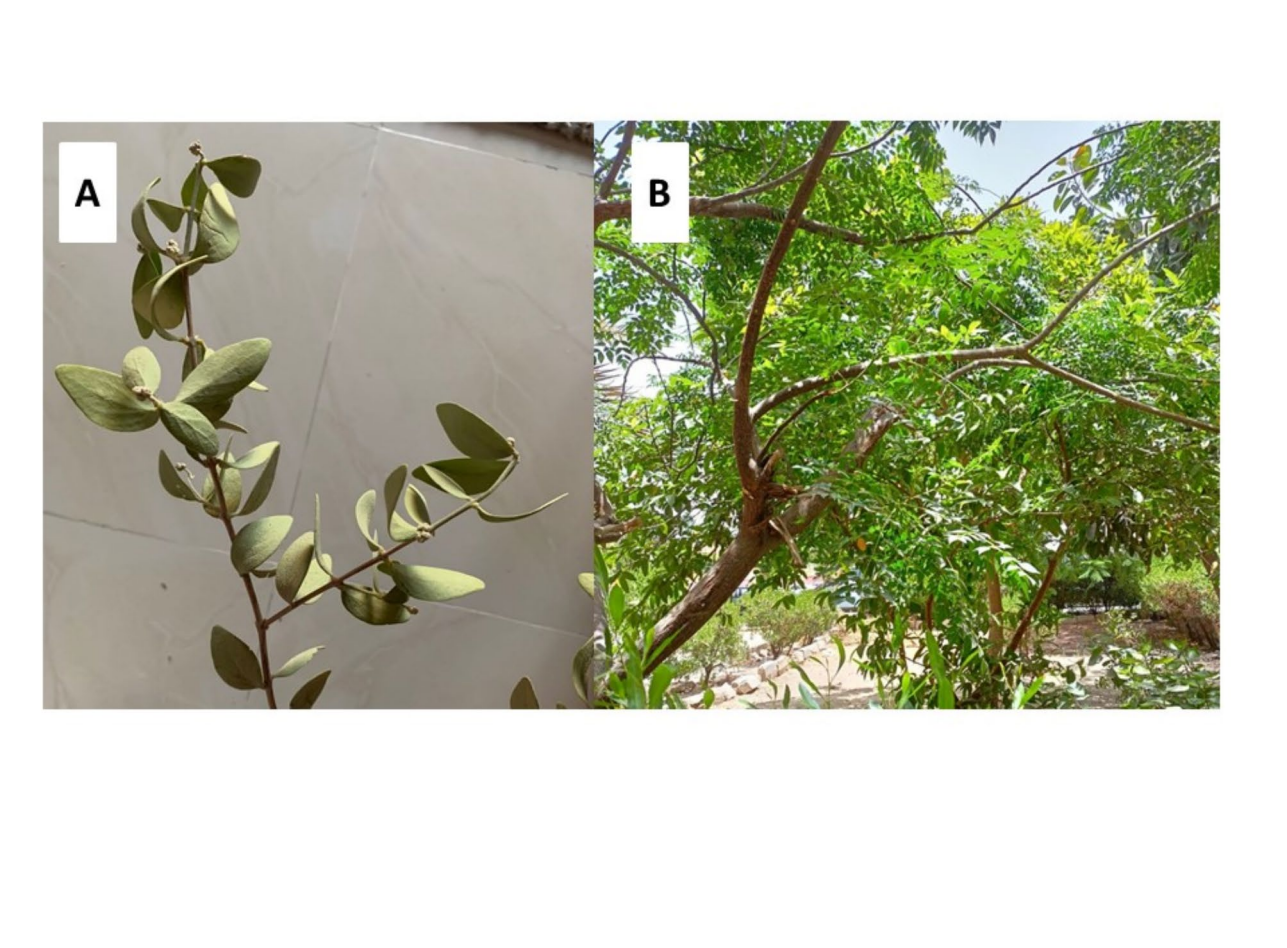
Morphology of (**a**) *Azadirachta indica* and (**b**) *Simmondsia chinensis.* The plant materials used were *Simmondsia chinensis* and *Azadirachta indica* leaves, which were purchased from Wadi El-Natron Valley, Beheira Governorate, 150 km northwest of Cairo (Lat. = 30° 30’ N, Long. = 30° 09’ E & Elev. = 30 m), Egypt.

The plant materials of *Azadirachta indica* and *Simmondsia chinensis* used in this study (Table 4) were purchased from local markets, Beheira Governorate, 150 km northwest of Cairo (Lat. = 30° 30’ N, Long. = 30° 09’ E & Elev. = 30 m), Egypt as provided in the ethical approval. The experiment complied with relevant institutional, national, and international guidelines and legislation. The collected plants as shown in Fig. 14 were cleaned, disinfected, and then rinsed with distilled water before being dried in the shade. The dried plant material was ground into a fine powder and passed through a 100 mm sieve.

**Table 4:** The ethnobotanical data of the employed plant species.

Plant species	Family	Common name	Plant part
*Simmondsia chinensis*	*Simmondsiaceae*	Jojoba	Stem, leaves and fruits
*Azadirachta indica*	*Meliaceae*	Neem	Stem and leaves

### Preparation of plant extracts

Ethanolic Jojoba extract was made as follows: 50 g of air-dried Jojoba powder was mixed with 100 ml of ethanol (95%), stored in a sterile flask, and shaken at 190–220 rpm for 24 h.

The extract was filtered and centrifuged for 15 min at 5000 rpm. Membrane filtering equipment was used to sterilize the supernatant. When not in use, the produced filtrate was placed in sterile bottles and preserved at 4 °C in a refrigerator^26^.

Fresh Neem leaves were washed in running tap water, dried, and kept in the dark for 5–7 days. The dried leaves were processed to create a fine powder (50 g), which was then mixed with 100 mL of each ethanol mixture for 24 h^79,80^.

The prepared extracts were further sterilized by passing through a 0.45 μm pore-size filter. Then, the purity of the extracts was checked by adding 2 ml of each extract to 10 ml of sterile nutrient broth and incubated at 37 °C for 24 h. Extract sterility was confirmed by the absence of broth turbidity after the incubation period^81^.

### Biosynthesis of CuO NPs by using plant extracts

For the CuO NP bioreduction reaction, 5 ml of plant extract (ethanlic jojoba or ethanolic neem extract) was mixed with 45 ml of 1 mM CuSO4.5H2O (Sigma‒Aldrich, St. Louis, MI, USA) solution, and the mixture was constantly stirred for 24 h at 30 °C and pH = 8. The resultant CuO NP solutions were centrifuged for 15 min at 12,000 rpm for 3 h. The CuNPs were dried at 80 °C in a hot air oven^82^.

### Characterization of the biosynthesized CuO NPs

The biosynthesized CuO NPs were characterized via TEM, UV‒vis spectroscopy, EDX, and FTIR spectroscopy^83^.

### TEM measurements

For TEM analysis, a drop of the solution was placed on carbon-coated copper grids (CCGs) and dried by allowing water to evaporate at room temperature. Electron micrographs were obtained using a JEOL JEM-1010 transmission electron microscope at 80 kV at The Regional Center for Mycology and Biotechnology (RCMB) Al-Azhar University^84^. This technique was carried out at The Regional Center for Mycology and Biotechnology (RCMB) Al-Azhar University using TEM (JEOL JEM-1010) to obtain the size, surface, shape, crystal structure and morphological data of the biosynthesized nanoparticles.

### UV‒vis spectroscopic analyses

One milliliter of biosynthesized NPs was scanned to detect the presence of specific peaks for copper NPs using UV‒vis spectroscopy (SPECTRO starnano BMG Labtech, Germany). This technique was carried out at the Central Lab, Ain shams University, Cairo, Egypt. The optical absorbance spectra were all recorded at similar intervals of wavelength (0.1 nm), with light wavelengths ranging from 300 to 800 nm. To establish the baseline, the samples were diluted 1:1 with double-distilled water^85^.

### FTIR spectroscopy analyses

The vibration and rotation of molecules influenced by infrared radiation at a particular wavelength were measured using FTIR^86^. This technique was used at the National Research Centre, Cairo, Egypt, to identify the functional groups of the active compounds according to the peak value in the region of the infrared region using an FTIR-ATR Brucker Vertex 80 V. The pellets were scanned at 4 cm^− 1^ resolution in the spectral range of 4000 to 400 cm^− 1^ at room temperature.

### EDX analyses

EDX was utilized for quantitative elemental analysis and was carried out at the National Research Centre using high-resolution SEM (model Quanta 250 FEG; FEI-Co., Netherlands). Approximately 3 mL of the CuO NP-containing solution was utilized for the EDX study, which allowed the various element energy absorption patterns to be observed^87^.

### Antibacterial activity of CuO NPs

The antibacterial activity of the biosynthesized CuO NPs was analyzed according to the Kirby–Bauer (agar disc diffusion) method^88^. The inhibitory activity was tested against 55 clinical bacterial pathogens as indicated in methodology Sect. 2. One hundred microliters of each fresh culture was spread on a nutrient agar plate with a sterile cotton swab. The paper disk was immersed in each sample of biosynthesized CuO NPs for 10 s. Then, the discs were placed into Petri-dishes inoculated with the tested pathogenic isolates and incubated at 37 °C for 24 h. The plates were incubated for 2 h in a refrigerator to allow diffusion of the extract into the medium. The antibacterial activity was indicated by measuring the inhibition zone (mm) around the paper disk.

### Determination of the minimum inhibitory concentration (MIC) and minimum bactericidal concentration (MBC) of CuO NPs

The initial concentration (1000 µg/ml) was diluted using double-fold serial dilution by adding 100 µl of CuO NP sample to 100 µl of sterile nutrient broth loaded in polystyrene sterile flat-bottomed 96-well plates to obtain a 500 µg/ml concentration. The above process was repeated several times to obtain concentrations of 250 µg/ml, 125 µg/ml, 62.5 µg/ml and 31.25 µg/ml^89^. After each concentration, 5 µl of the standardized bacterial suspension was inoculated (55 bacterial isolates as indicated in Methodology Sect. 2), and the mixture was incubated at 37 °C for 24 h. The plates were read by an ELISA reader. The growth of the inoculum in broth was indicated by broth turbidity, and the lowest concentration of the CuNPs that inhibited the growth of the test organism was taken as the MIC. The negative control referred to the nutrient broth, and the NP positive control was performed using nutrient broth and the tested pathogen.

For MBC detection, streaks from the lowest concentrations of CuO NPs that showed no noticeable growth were spread out on Mueller–Hinton agar plates. Following a 24-hour incubation period at 37 °C, the plates were examined for bacterial growth in accordance with the concentrations of the plant extract and CuO NPs. The newly inoculated agar plates that did not show any bacterial growth had the lowest concentration of NPs, which was determined as the MBC^90^.

The tolerance level of CuO NPs was determined for each strain by using the following formula: MBC/MIC.

Two activities were illustrated by the NPs: bacteriostatic (MBC/MIC ≥ 4) and bactericidal (MBC/MIC < 4)^91^.

### Biofilm inhibition assay of CuO NPs

Clinical isolates described in methodology Sect. 2 were incubated overnight in tryptone soy broth (TSB) containing 0.25% glucose. Then, a 0.5 McFarland (1:100) concentration of this fresh culture was transferred to 96-well polystyrene microtiter plates. Then, 100 µL of diluted biosynthesized NPs was added to the wells and incubated at 37 °C for 24 h. Planktonic bacteria from each well were washed 3 times with sterile phosphate-buffered saline (PBS) and dried. Then, 200 µL of a 0.1% crystal violet solution was added to the wells for 15 min. After washing three times and drying, 200 µL of 30% glacial acetic acid was added to the wells. The absorbance was measured at 570 nm after a 15-minute, room-temperature incubation^92^.

### Antioxidant activity assay of CuO NPs

The antioxidant activity of the CuO NPs was tested using a 2,2-diphenylpicrylhydrazyl (DPPH) assay^93^. An aliquot of a 0.1 mM solution of DPPH was prepared, ranging from 1000 µg/ml to 3.9 µg/ml. One milliliter of DPPH solution was added to 3 ml of sample at each concentration (1000, 500, 250, 125, 62.5, 31.25, 15.62, 7.81, and 3.9 µg/ml), and the mixture was shaken and incubated at room temperature for 30 min. The absorbance was subsequently measured at O.D. 517 nm using a spectrophotometer. The results were calculated as the IC50, which was considered to be the concentration at which 50% of the DPPH activity was lost. The DPPH scavenging effect (%) was measured using the following formula: DPPH scavenging effect (%) = [A_0_ − A_1_/A_0_ × 100].

where A_0_ = the control reaction and A_1_ = the OD of the sample.

### Cytotoxicity of CuO NPs against the HFB4 cell line

The cytotoxic effects of CuO NPs on a normal human melanocyte cell line (HFB4) were quantified by determining the 50% cell growth inhibition (IC50) by the MTT (3-(4,5-dimethylthiazol-2-yl)-2,5-diphenyl-2 H-tetrazolium bromide) assay^94^ at the Scienceway Centre, Cairo, Egypt. A 96-well tissue culture plate was inoculated with 1 × 10^5^ cells/ml (100 µl/well) and incubated at 37 °C for 24 h to develop a complete monolayer sheet. The growth medium (Dulbecco’s modified Eagle medium [DMEM]) was discarded from the plates after a confluent sheet of cells had formed, and the cell monolayer was washed twice with wash media. Then, two-fold dilutions of the tested samples were subsequently made in Rosewell Park Memorial Institute (RPMI) medium supplemented with 2% serum (maintenance medium). An aliquot of 20 µl of MTT solution was added to each well, which was subsequently placed on a shaking table at 150 rpm for 5 minutes to thoroughly mix the MTT solution with the media. Then, the mixture was incubated at 37 °C and 5% CO2 for 4 h to allow the MTT to be metabolized. Later, the media was removed, and the cells were resuspended in formazan (the MTT metabolic product) dissolved in 200 µl of DMSO. The plate was placed on a shaking table at 150 rpm for 5 min to thoroughly mix the formazan in the solvent. The optical density was recorded at 560 nm. The optical density should be directly correlated with the quantity of living cells.

### Determination of the bactericidal mechanism of the biosynthesized CuO NPs formed from the ethanolic Jojoba extract

One milliliter of MRSA and *Klebsiella pneumoniae* cultures at the stationary growth phase were treated with the minimum inhibitory concentration (MIC) of the biosynthesized CuO NPs for 1 h. The unabsorbed nanoparticles were extracted from the treated samples by centrifuging them for 2 min at 2,000 rpm. After being cleaned with distilled water and centrifuged once more for 25 min at 15,000 rpm, the pellet was dissolved in 0.5 ml of distilled water. One drop of the bacterial cell suspension was added to carbon-coated grids (80 mesh square grid, EMS, TED PELLA, Inc., Redding, CA, USA) and incubated for 2 min. Blotting paper was used to remove the extra sample from the grid. After 5 µL of 1% uranyl acetate was applied to the grid, the samples were stained and left for one minute. For the control sample, one milliliter of MRSA and *Klebsiella pneumoniae* culture in the stationary growth phase was centrifuged once more for 25 min at 15,000 rpm, after which the pellet was dissolved in 0.5 ml of distilled water. One drop of the bacterial cell suspension was added to the carbon-coated grids and incubated for 2 min. Blotting paper was used to remove the extra sample from the grid. After 5 µL of 1% uranyl acetate was applied to the grid, the samples were stained and left for one minute. Then, transmission microscopy (JEOL JEM-1010 at 80 kV) was conducted at The Regional Center for Mycology and Biotechnology (RCMB) Al-Azhar University, Cairo, Egypt^36^.

### Molecular docking

Molecular docking, a well-known and adaptable in silico technique, allows for the discovery of biologically pertinent models from compound set manufacture. The method predicts how different ligands’ optimum conformers will interact with the receptor protein, aiding in the selection of synthetic targets. We carried out molecular docking analyses of CuO-NPs using Molecular Orbital Environment (MOE) software to investigate the binding modes between the ligand and the targeted enzymes (crystal structure of penicillin-binding protein 4 (PBP4) from *Staphylococcus aureus* (PDB ID: 1TVF) and crystal structure of OXA-48 beta-lactamase (PDB ID: 7AUX)).

The compound structure was modelled using the Gaussian 09 program^95^ and exported as MOL files (.mol) for MOE appearance.

The crystal structures of penicillin-binding protein 4 (PBP4) and OXA-48 beta-lactamase were obtained from the protein data bank (http://www.rcsb.org/pdb, accessed on 14 April 2024). Hydrogen atoms were included after the water molecules around the protein had been eliminated. The parameters and charges were determined using the MMFF94x force field. After creating alpha-site spheres using the MOE site finder module, we docked our compound in the active site using the MOE DOCK module. The dock score for the MOE program was determined using the London dG scoring method, with placement as a triangle matcher, retention as 10, and refinement as a force field. The leading conformations of the docked ligands were identified by considering the RMSD values, binding energies, and binding modes of the selected residues^96^.

### Statistical analysis

The results are shown as the mean ± standard deviation (SD) and were analyzed using Microsoft Excel 2013, Analysis Toolpack (Microsoft Corporation). The standard errors for each graph were plotted after all the data were calculated from a minimum of duplicate data.

## Conclusion

In this study, copper oxide nanoparticles were synthesized in a green way by using ethanolic extracts of plants. This method of CuO NP production has several benefits, including simplicity and the ability to scale up the process economically. The presence of these biosynthesized CuO NPs was confirmed by UV‒Vis spectroscopy (at 344 nm), TEM, FTIR, and EDX, and the biosynthesized CuO NPs demonstrated strong antibacterial and antibiofilm activities against a variety of multidrug-resistant bacteria. Finally, the cytotoxic effect of the biosynthesized CuO NPs against HBF4 was observed for the safe use of CuO NPs in the biomedical field, and CuO NPs could be promising antibiotic alternatives. By all counts, and with proven results it is no wonder that biosynthesized copper oxide nanoparticles (CuO NPs) have a wide range of promising future applications across various fields. They are being explored for use in drug delivery systems, wound healing, and as antibacterial agents^1^. In addition, Their catalytic properties make them suitable for various industrial processes, including the synthesis of chemicals and the degradation of environmental pollutants.

## Electronic supplementary material

Below is the link to the electronic supplementary material.

Supplementary Material 1

## Data Availability

All data generated or analysed during this study are included in this published article and its supplementary information files.
