# Supplementary material for: Antibacterial activity of green synthesized copper oxide nanoparticles against multidrug-resistant bacteria
Source: Sci Rep. 2024 Oct 23;14:25020. doi: 10.1038/s41598-024-75147-0 (PMC11499942; doi:10.1038/s41598-024-75147-0)
Supplement: Supplementary file 1 — Supplementary Material 1 [file 41598_2024_75147_MOESM1_ESM.docx]

**Supplementary table 1: Antibacterial activity of biogenic CuO NPs against tested isolates**

| **Isolate** | **Inhibition zone diameter (mm)** | |
| --- | --- | --- |
|  | **CuO NPs biosynthesized by** | |
|  | **Ethanolic Neem extract** | **Ethanolic Jojoba extract** |
| ***Acinetobacter spp.*** | 34 ± 4.24 | 28 ± 2.82 |
| **MRSA** | 32 ± 2.12 | 28 ± 2.12 |
| ***E. coli*** | 24 ± 3.53 | 23 ± 0.70 |
| ***Klebsiella spp*** | 35 ± 2.82 | 24 ± 2.82 |
| ***Pseudomonas spp.*** | 27 ± 3.53 | 19 ± 2.12 |
| ***Stenotrophomonas spp.*** | 30 ± 2.12 | 30 ± 0.70 |

**Supplementary table 2: Antibiofilm activity of biosynthesized CuO NPs against bacterial strains**

| **Bacterial isolates** | **Concentrations (μg/ml)** | | | | | | |
| --- | --- | --- | --- | --- | --- | --- | --- |
|  | **CuO NPs biosynthesized by ethanolic Neem extract** | | | | | | |
|  | **500** | **250** | **125** | **62.5** | **31.25** | **15.62** | **7.81** |
| ***E. coli*** | 97.7 | 93 | 92.9 | 92 | 88.7 | 73.8 | 72.4 |
| ***Klebsiella pneumoniae*** | 98 | 98.5 | 95.6 | 94.7 | 94 | 93 | 34.5 |
| ***Acinetobacter* spp** | 95 | 93.9 | 93.5 | 90.5 | 87.9 | 85.8 | 84.1 |
| **MRSA** | 81.1 | 75.1 | 74.2 | 62.2 | 57.5 | 51.9 | 53.6 |
| **CuO NPs biosynthesized by ethanolic Jojoba Extract** | | | | | | | |
| ***E. coli*** | 93.3 | 91.1 | 90.2 | 89.2 | 72.8 | 67.7 | 67.2 |
| ***Klebsiella pneumoniae*** | 99.1 | 97.5 | 95 | 93.7 | 86.6 | 81.5 | 79.8 |
| ***Acinetobacter* spp** | 95.2 | 94.8 | 91.5 | 91.4 | 88.5 | 88.3 | 72.1 |
| **MRSA** | 95.7 | 93.8 | 93.4 | 87.2 | 75.9 | 68.8 | 63.2 |

**Supplementary table 3: MTT assay using different concentrations of CuO NPs biosynthesized by ethanolic Neem extract treated HBF-4 cells.**

| Conc. (μg/mL) | Mean | SD | Viability % |
| --- | --- | --- | --- |
| 500 | 0.212333 | 0.004631 | 28.73252143 |
| 250 | 0.668333 | 0.00786 | 90.43752819 |
| 125 | 0.737 | 0.001 | 99.72936401 |
| 62.5 | 0.738 | 0.002517 | 99.864682 |
| 31.25 | 0.738667 | 0.002404 | 99.954894 |
| 15.62 | 0.735333 | 0.002186 | 99.50383401 |

**Supplementary table 4: MTT assay using different concentrations of CuO NPs biosynthesized by ethanolic Jojoba extract treated HBF-4 cells.**

| Conc. (μg/mL) | Mean | SD | Viability % |
| --- | --- | --- | --- |
| 500 | 0.175333 | 0.006119 | 23.72575553 |
| 250 | 0.635333 | 0.00491 | 85.97203428 |
| 125 | 0.733333 | 0.003528 | 99.23319802 |
| 62.5 | 0.737667 | 0.00348 | 99.819576 |
| 31.25 | 0.733667 | 0.001202 | 99.27830401 |
| 15.62 | 0.738667 | 0.001453 | 99.954894 |

**Conc.:** conentrations**; SD**: standard Deviation

| 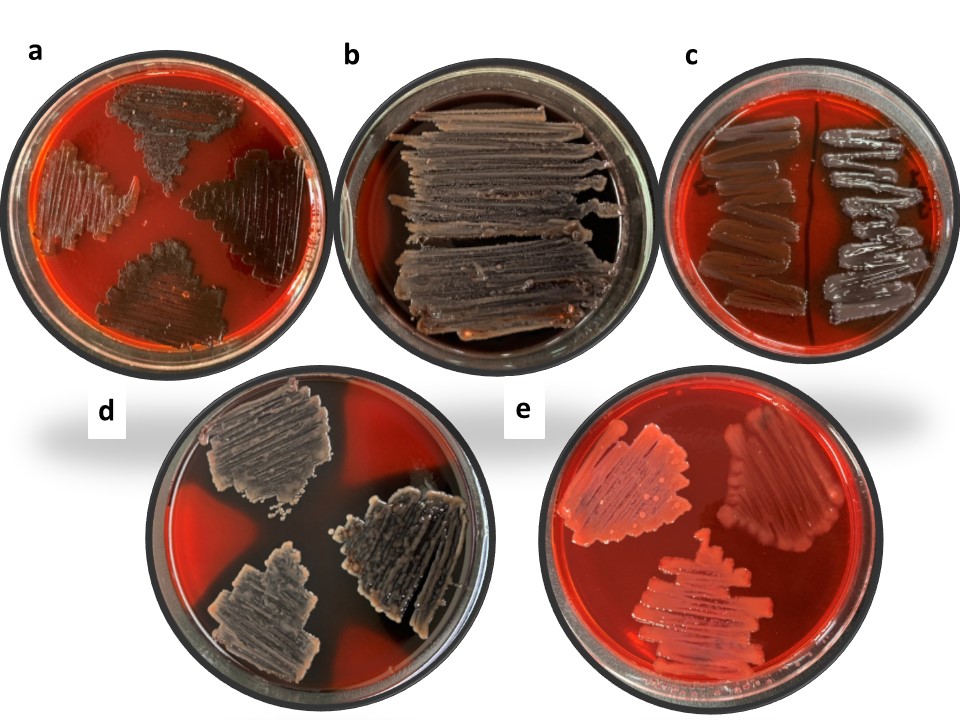 |
| --- |
| **Supplementary figure 1: Screening of biofilm production for (a) *Acinetobacter spp.,* (b) *K. pneumoniae,* (C) *E. coli,* (d) MRSA, and (e) *Pseudomonas aeruginosa.*** all bacterial strains inoculated on Congo agar plates, then incubated at 37 °C for 24 h, biofilm producers bacteria appear as black colonies with a dry crystalline consistency while non-biofilm producers bacteria remained pink colonies. |

| **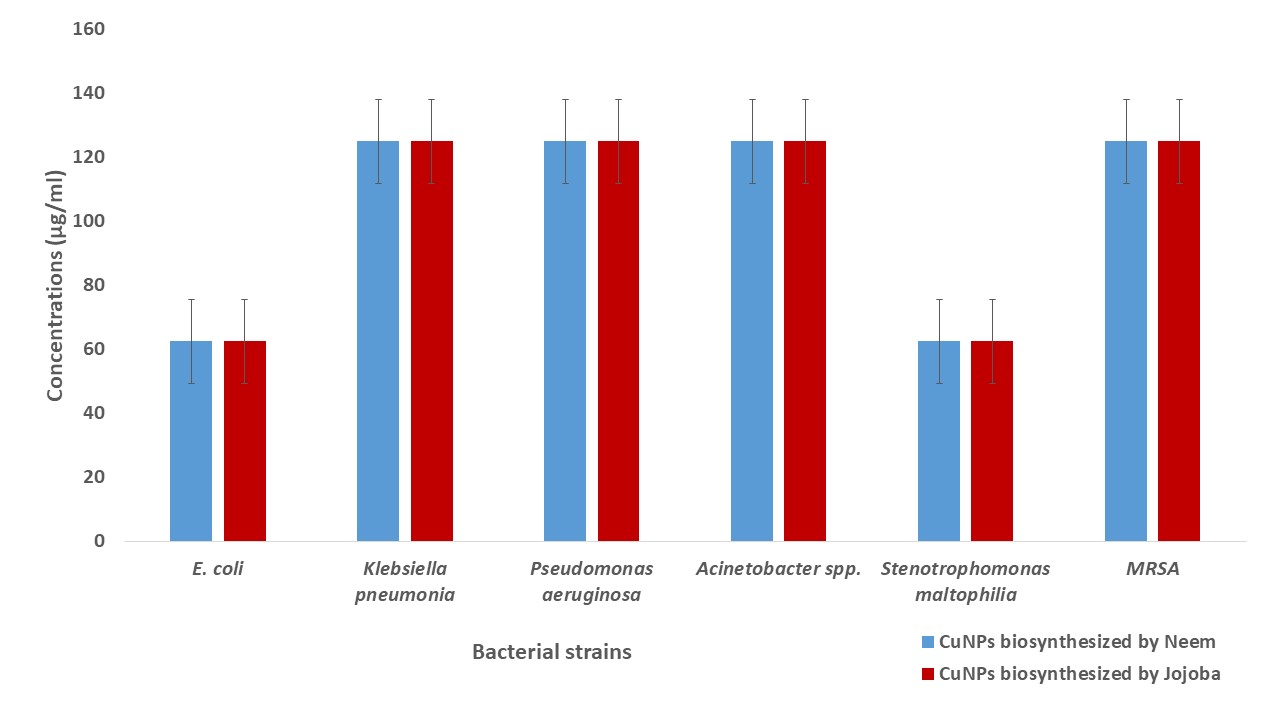** |
| --- |
| **Supplementary figure 2: MIC of biosynthesized CuO NPs against bacterial strains at different concentrations.** MIC of biosynthesized CuO NPs measured by broth dilution method against bacterial strains, initial concentration (1000µg/ml) was diluted using double fold serial dilution by adding 100µl CuO NPs samples to100µl sterile nutrient broth. Each concentration inoculated in duplicates with with 5µl of the standardized bacterial suspension and incubated at 37 °C for 24 h. |
